# ALKBH3‐Mediated M^1^A Demethylation of METTL3 Endows Pathological Fibrosis:Interplay Between M^1^A and M^6^A RNA Methylation

**DOI:** 10.1002/advs.202417067

**Published:** 2025-02-28

**Authors:** Liying Tu, Shuchen Gu, Ruoqing Xu, En Yang, Xin Huang, Hsin Liang, Shenying Luo, Haizhou Li, Yixuan Zhao, Tao Zan

**Affiliations:** ^1^ Department of Plastic and Reconstructive Surgery Shanghai Ninth People's Hospital Shanghai Jiao Tong University School of Medicine 639 Zhizaoju Road Shanghai 200011 P. R. China

**Keywords:** ALKBH3, fibroblast, METTL3, pathological skin fibrosis, RNA methylation

## Abstract

Epigenetic modifications serve as crucial molecular switches for pathological fibrosis; howbeit the role of m^1^A in this condition remains enigmatic. Herein, it is found that ALKBH3 exerts a pro‐fibrotic effect in pathological skin fibrosis by reshaping N6‐methyladenosine (m^6^A) RNA modification pattern. First, ALKBH3 exhibited specific upregulation within hypertrophic scars (HTS), accompanied by N1‐methyladenosine (m^1^A) hypomethylation. Moreover, multiomics analyses identified METTL3, a critical writer enzyme involved in m^6^A modification, as a downstream candidate target of ALKBH3. Therapeutically, ablation of ALKBH3 inhibited the progression of HTS both in vitro and in vivo, while exogenous replenishment of METTL3 counteracted this antifibrotic effect. Mechanistically, ALKBH3 recognizes the m^1^A methylation sites and prevents YTHDF2‐dependent mRNA decay of METTL3 transcript. Subsequently, METTL3 stabilizes collagen type I alpha 1 chain (*COL1A1*) and fibronectin1 (*FN1*) mRNAs, two major components of extracellular matrix, and therefore eliciting the pathological transformation of HTS. This observation bridges the understanding of the link between m^1^A and m^6^A methylation, the two fundamental RNA modifications, underscoring the participation of “RNA methylation crosstalk” in pathological events.

## Graphical abstract

RNA modification is crucial in fibrosis diseases, but the role of m^1^A modification remains elusive. This study reveals that ALKBH3, an m^1^A demethylase, drives skin fibrosis by reshaping m^6^A RNA modification and stabilizing *COL1A1* and *FN1* mRNAs through METTL3, uncovering a crosstalk between m^1^A and m^6^A methylation in pathological events.

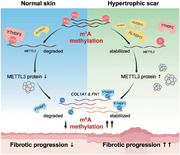

## 1 Introduction

A series of cutaneous fibroproliferative diseases, including hypertrophic scar (HTS), keloid, and scleroderma, are clinically characterized by skin fibrosis.^[^ ^1^, ^2^ ^]^ Skin fibrosis is a process characterized by excessive proliferation of fibroblasts and abnormal deposition of extracellular matrix (ECM) and is often accompanied by intolerable itching, contracture deformities, and functional impairments.^[^ ^3^ ^]^ Considering its effects on physical, mental, and social health, skin fibrosis is increasingly becoming recognized as one of today's major health‐care challenges.^[^ ^4^ ^]^ However, therapeutic results for skin fibrosis frequently fall short of expectations.^[^ ^5^ ^]^ The Transforming Growth Factor‐β/Smad (TGF‐β/Smad) signaling pathway, the canonical pathway mediating skin fibrosis,^[^ ^5^, ^6^ ^]^ participates in diverse biological processes across multiple organs and systems.^[^ ^7^ ^]^ The broad involvement of this pathway makes the identification of specific therapeutic targets challenging. Hence, exploring novel pathogenic mechanisms and identifying therapeutic targets for skin fibrosis are highly important.

N1‐methyladenosine (m^1^A) is an important posttranscriptional RNA modification identified in transfer RNAs (tRNAs), ribosomal RNAs (rRNAs), and messenger RNAs (mRNAs).^[^ ^8^, ^9^ ^]^ In mRNAs, m^1^A has been detected in every segment and functions as a unique type of base methylation to block Watson–Crick base pairing and alter mRNA structural stability^[^ ^10^, ^11^ ^]^ The dynamics of m^1^A methylation are mediated by methyltransferases (“writers”: TRMT6, TRMT61A, TRMT61B, and TRMT10C), demethylases (“erasers”: ALKBH1, ALKBH3, and FTO) and RNA‐binding proteins (“readers”: YTHDFs).^[^ ^12^ ^]^ m^1^A modification has recently been recognized to play a crucial role in RNA regulation, thus participating in various biological processes, such as gene expression, RNA stability regulation, posttranscriptional regulation, and disease occurrence.^[^ ^13^ ^]^ Notably, multiple RNA modifications are involved in the pathogenesis of skin fibrosis. For instance, N6‐methyladenosine (m^6^A) hypermethylation may contribute to keloid pathogenesis by regulating the Wnt/β‐catenin pathway.^[^ ^14^ ^]^ However, the regulatory roles of m^1^A, another important form of RNA modification, in skin fibrosis remain to be fully addressed.

Thus, we aimed to identify the molecular mechanisms and clinical potential of m^1^A modification in skin fibrosis. Our study reveals for the first time that the decreased global m^1^A level, driven by elevated ALKBH3 expression, are a key feature of skin fibrosis. Additionally, by using RNA sequencing (RNA‐seq) and methylated RNA immunoprecipitation–sequencing (MeRIP‐seq), we found that ALKBH3 upregulates methyltransferase‐like 3 (METTL3) expression via YTHDF2. The increased METTL3 stabilized *COL1A1* and *FN1* mRNAs through YTHDF1‐mediated m^6^A modification, thereby advancing pathological skin fibrosis. Therapeutically, silencing ALKBH3 exhibited promising effects on skin fibrosis both in vitro and in vivo, which were diminished when METTL3 expression was restored, suggesting a novel strategy for treating skin fibrosis. Importantly, this study establishes a link between m^1^A and m^6^A methylation, the two fundamental RNA modifications, underscoring the participation of a “RNA methylation crosstalk” in pathological events.

## 2 Results

### 2.1 Reduced RNA m^1^A Modification and Elevated ALKBH3 Expression are Observed in Hypertrophic Scars

**Figure 1:**
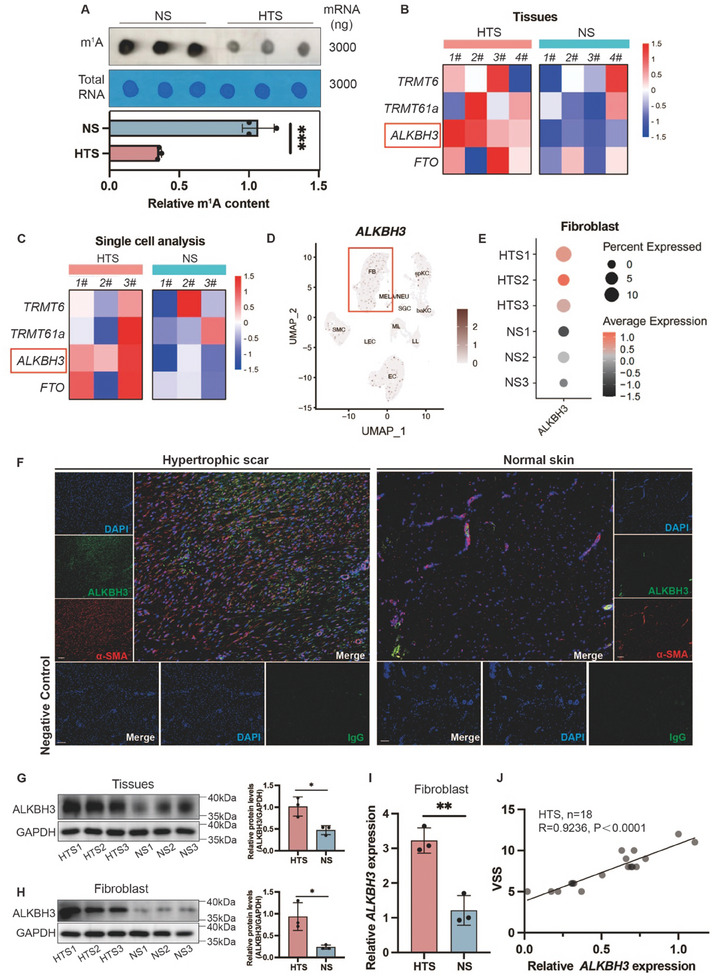
Reduced RNA m^1^A modification and elevated ALKBH3 expression were observed in hypertrophic scars. A) Dot blot showing the m^1^A signal relative to the methylene blue signal in HTS and NS samples. The data are presented as the mean ± SD of triplicate experiments. Significance was determined by unpaired two‐tailed Student's t test. ^***^ *p* < 0.001. B) Heatmap of m^1^A writer and eraser gene expression in HTS and NS samples. The data are representative of triplicate experiments. C) Heatmap of m^1^A writer and eraser gene expression in HTS and NS samples according to single‐cell sequencing analysis (GSE156326). D) According to single‐cell sequencing analysis, ALKBH3 is localized primarily in fibroblasts. E) Single‐cell sequencing analysis revealed greater expression of ALKBH3 in HDFs than in NDFs. F) Immunofluorescence of ALKBH3 (green), α‐SMA (red), and DAPI (blue) in HTS and NS samples. Scale bars: 100 µm. G) Western blot showing ALKBH3 expression relative to GAPDH in HTS and NS samples. The data are presented as the mean ± SD of triplicate experiments. Significance was determined by unpaired two‐tailed Student's t test. ^*^ *p *< 0.05. H) Western blot showing ALKBH3 expression relative to GAPDH in fibroblasts isolated from HDFs and NDFs. The data are presented as the mean ± SD of triplicate experiments. Significance was determined by unpaired two‐tailed Student's t test. I) qRT‒PCR data showing *ALKBH3* expression in HDFs and NDFs samples. The data are presented as the mean ± SD of triplicate experiments. Significance was determined by unpaired two‐tailed Student's t test. J) Correlation analysis between *ALKBH3* expression and the VSS score. *GAPDH* was used to normalize mRNA expression levels, and the 2^−△△Ct^ method was used to calculate the relative expression levels (*n* = 18, R = 0.9236, *p* < 0.0001). The *ALKBH3* expression level in sample HTS1 was defined as 1, and the expression levels were normalized to the fold changes. VSS, Vancouver Scar Scale; HTS, hypertrophic scar; NS, normal skin; HDF, hypertrophic scar derived fibroblast; NDF, normal skin derived fibroblast.

Hypertrophic scars (HTS), which are typical manifestations of skin fibrosis, were chosen as a model to elucidate the role of m^1^A in pathological skin fibrosis. We first compared the global m^1^A modification level between HTS and normal skin (NS). Notably, HTS samples exhibited significantly lower m^1^A levels, as demonstrated by the m^1^A dot blot assay (**Figure** **1**A). Furthermore, we analyzed the expression of m^1^A writers and erasers, and we observed significant upregulation of *ALKBH3* mRNA expression in HTSs, while the expression of other regulators remained unchanged (Figure 1B). This finding was further supported by the results of a previous single‐cell analysis (GSE156326),^[^ ^15^ ^]^ which also revealed that the increase in *ALKBH3* expression was predominantly localized to fibroblasts (Figure 1C–E). Although ALKBH3 expression was also detected in endothelial cells (ECs) and smooth muscle cells (SMCs) (Figure 1D), no significant difference in expression was observed between HTS and NS in these cell types (Figure S1A, Supporting Information). Moreover, ALKBH3 had no significant impact on the phenotypes of ECs (Figure S1B–G, Supporting Information) or SMCs (Figure S1H–L, Supporting Information). Therefore, the increased expression of ALKBH3 in fibroblasts remains a critical driving factor in pathological skin fibrosis. Immunofluorescence (IF) staining and western blot (WB) analyses revealed elevated protein expression of the demethylase ALKBH3 in HTSs (Figure 1F,G), which corresponded with a reduction in the m^1^A levels in the HTS. The trend was further confirmed in fibroblasts isolated from HTS tissues (Figure 1H,I). Moreover, high ALKBH3 expression was significantly correlated (R = 0.9236, *p* value < 0.0001) with more advanced HTS stages, as assessed by the modified Vancouver Scar Scale (mVSS) (Table S1, Supporting Information), underscoring the functional importance of ALKBH3 in HTS (Figure 1J).

### 2.2 Inhibition of ALKBH3 Attenuates HTS Derived Fibroblasts Activation In Vitro

**Figure 2:**
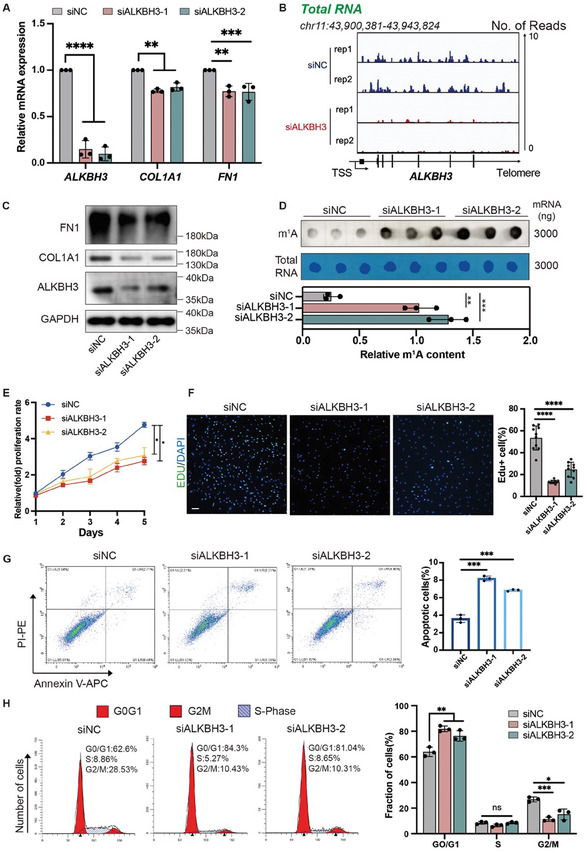
Inhibition of ALKBH3 in HDFs attenuates fibroblast activation in vitro. A) qRT‒PCR data showing *ALKBH3, COL1A1 and FN1* expression in HDFs upon ALKBH3 knockdown. The data are presented as the mean ± SD of triplicate experiments. Significance was determined by unpaired two‐tailed Student's t test. ^**^ *p* < 0.01, ^***^ *p* < 0.001, ^****^ *p* < 0.0001. B) IGV tracks from the m^1^A‐MeRIP‐seq analysis show decreased *ALKBH3* expression in HDFs upon ALKBH3 knockdown. C) Western blot showing ALKBH3, COL1A1, and FN1 expression relative to GAPDH in HDFs upon ALKBH3 knockdown. D) Dot blot showing the m^1^A signal relative to the methylene blue signal in HDFs upon ALKBH3 knockdown. The data are presented as the mean ± SD of triplicate experiments. Significance was determined by unpaired two‐tailed Student's t test. ^**^ *p* < 0.01, ^***^ *p* < 0.001. E) A CCK‐8 assay was used to evaluate the proliferation of HDFs upon ALKBH3 knockdown. The data are presented as the mean ± SD of triplicate experiments. Significance was determined by unpaired two‐tailed Student's t test. ^*^ *p* < 0.05. F) An EdU (green) incorporation assay was employed to evaluate the proliferation of HDFs upon ALKBH3 knockdown. Scale bar: 100 µm. The data are presented as the mean ± SD of triplicate experiments, and ten random fields were included in the analysis. Significance was determined by unpaired two‐tailed Student's t test. ^****^ *p* < 0.0001. G) Apoptosis in cells with ALKBH3 knockdown was analyzed by flow cytometry. All of the experiments were performed in triplicate. Significance was determined by unpaired two‐tailed Student's t test. ^***^ *p* < 0.001. H) Cell cycle distribution of HDFs following ALKBH3 knockdown. All of the experiments were performed in triplicate. Significance was determined by unpaired two‐tailed Student's t test. ^*^ *p *< 0.05, ^**^ *p *< 0.01, ^***^ *p *< 0.001. HDF, hypertrophic scar derived fibroblast.

To explore the biological function of ALKBH3 in fibroblasts, we utilized two individual small interfering RNAs (siRNAs) to silence ALKBH3 in HTS derived fibroblasts (HDF) (**Figure** **2**A–C; Figure S2, Supporting Information). Concordantly, ALKBH3 knockdown led to a marked increase in m^1^A levels in HDFs (Figure 2D). Furthermore, a reduction in collagen deposition capacity was observed in ALKBH3‐silenced HDFs, as demonstrated by quantitative reverse transcription–PCR (qRT‒PCR) (Figure 2A) and WB (Figure 2C; Figure S2,Supporting Information) analyses. ALKBH3 knockdown also resulted in decreased cell proliferation, as evidenced by both Cell Counting Kit‐8 (CCK‐8) and 5‐ethynyl‐2′‐deoxyuridine (EdU) incorporation assays (Figure 2E,F). Moreover, flow cytometric analysis revealed an increase in apoptosis and alterations in the cell cycle distribution after ALKBH3 inhibition (Figure 2G,H). Notably, the knockdown of ALKBH3 resulted in negligible changes in the migration of HDFs (Figure S3, Supporting Information). Additionally, silencing ALKBH3 in normal dermal fibroblasts (NDFs) did not significantly affect collagen deposition, proliferation, or migration (Figure S4, Supporting Information). Taken together, these data suggest that ALKBH3 promotes pathological skin fibrosis primarily by driving the abnormally activated fibroblasts.

### 2.3 ALKBH3 Promotes the Progression of Skin Fibrosis In Vivo

**Figure 3:**
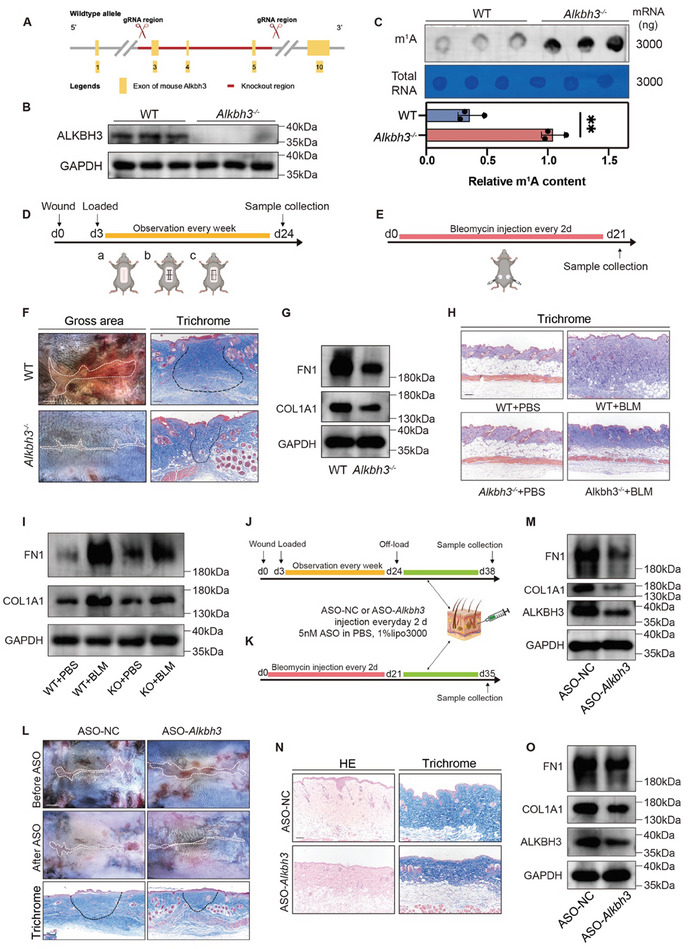
ALKBH3 knockout attenuates pathological skin fibrosis in vivo. A) Schematic diagram showing the strategy for *Alkbh3* gene deletion using CRISPR/Cas9 editing. B) The loss of ALKBH3 expression in the epidermis and dermis of *Alkbh3^‒/‒^* mice was confirmed by western blot analysis. C) Dot blot showing the m^1^A signal relative to the methylene blue signal in the skin of WT and *Alkbh3^‒/‒^* mice. The data are presented as the mean± SD of triplicate experiments. Significance was determined by unpaired two‐tailed Student's t test. ^**^ *p* < 0.01. D) Study design for the mechanical stretch‐induced HTS model (*n* = 6 biologically independent mice). E) Study design for the bleomycin‐induced skin fibrosis model (*n* = 6 biologically independent mice). F) Representative gross appearance (scale bar: 500 µm) and Masson staining images (scale bar: 100 µm) of scar tissue from mice in the mechanical stretch‐induced HTS model. G) Western blot showing COL1A1 and FN1 expression relative to GAPDH in the mechanical stretch‐induced HTS model. H) Representative Masson staining images from the bleomycin‐induced skin fibrosis model showing collagen deposition within scar tissue (scale bar: 100 µm). I) Western blot showing COL1A1 and FN1 expression relative to GAPDH in the bleomycin‐induced skin fibrosis model. J) Study design for treatment with ASO‐*Alkbh3* in the mechanical stretch‐induced HTS model (*n* = 6 biologically independent mice). K) Study design for treatment with ASO‐*Alkbh3* in the bleomycin‐induced skin fibrosis model (*n* = 6 biologically independent mice). L) Representative gross appearance (scale bar: 500 µm) and Masson staining images (scale bar: 100 µm) of scar tissue obtained from mice in the mechanical stretch‐induced HTS model following treatment with ASO‐*Alkbh3*. M) Western blot showing COL1A1 and FN1 expression relative to GAPDH in scar tissue obtained from mice in the mechanical stretch‐induced HTS model following treatment with ASO‐*Alkbh3*. N) Representative haematoxylin–eosin (HE) and Masson staining images from the bleomycin‐induced skin fibrosis model following treatment with ASO‐*Alkbh3* (scale bar: 100 µm). O) Western blot showing COL1A1 and FN1 expression relative to GAPDH in scar tissue obtained from mice in the mechanical stretch‐induced HTS model following treatment with ASO‐*Alkbh3*. HTS, hypertrophic scar; ASO, antisense oligonucleotide.

Based on the profibrotic role of ALKBH3 observed in vitro, we next established mice with global *Alkbh3* knockout (*Alkbh3^−/−^* mice) (**Figure** **3**A). The gross skin appearance and histological characteristics of these *Alkbh3^−/−^* mice were similar to those of their wild‐type (WT) littermates. The decrease in *Alkbh3* expression and increase in global m^1^A modification throughout the skin layers of *Alkbh3^−/−^* mice were verified through genotyping, WB analysis, IF staining, and a dot blot assay (Figure 3B,C; Figure S5, Supporting Information). We utilized mechanical stretch‐induced HTS and bleomycin‐induced skin fibrosis models to mimic pathological skin fibrosis in vivo (Figure 3D,E). In the mechanical stretch‐induced HTS model, the gross scar area was significantly attenuated in the *Alkbh3^−/−^* group (Figure 3F; Figure S6A, Supporting Information). In addition, collagen deposition in the dermis was also reduced in the *Alkbh3^−/−^* group, as revealed by qRT‒PCR and WB analyses (Figure 3G; Figure S6B,C, Supporting Information). Similarly, reductions in dermal thickness and collagen deposition were observed in *Alkbh3^−/−^* mice of bleomycin‐induced skin fibrosis model (Figure 3H,I; Figure S6D–F, Supporting Information).

To further elucidate the clinical implications of ALKBH3 inhibition in pathological skin fibrosis, we administered anti‐*Alkbh3* antisense oligonucleotides (ASOs) to WT mice after successful modeling (Figure 3J,K). ASOs are chemically modifiable single‐stranded nucleic acid sequences that function by binding to RNA sequences through Watson–Crick base pairing.^[^ ^16^ ^]^ ASOs designed to selectively downregulate, upregulate, or modify the expression of key genes in patients with terminal illnesses have become well‐established clinical therapeutic agents. Several ASO‐based drugs have received approval from the United States Food and Drug Administration (FDA), with numerous others currently undergoing clinical trials.^[^ ^17^ ^]^ Treatment with anti‐*Alkbh3* ASOs led to a significant reduction in the gross scar area, concomitant with reduced dermal thickness and collagen deposition in mice with pathological skin fibrosis (Figure 3L–O; Figure S7, Supporting Information). Taken together, these findings underscore the role of ALKBH3 in exacerbating pathological skin fibrosis progression in vivo, thus emphasizing its potential as a crucial therapeutic target.

### 2.4 Multiomics Screening Identified METTL3 as the Downstream Candidate of ALKBH3

**Figure 4:**
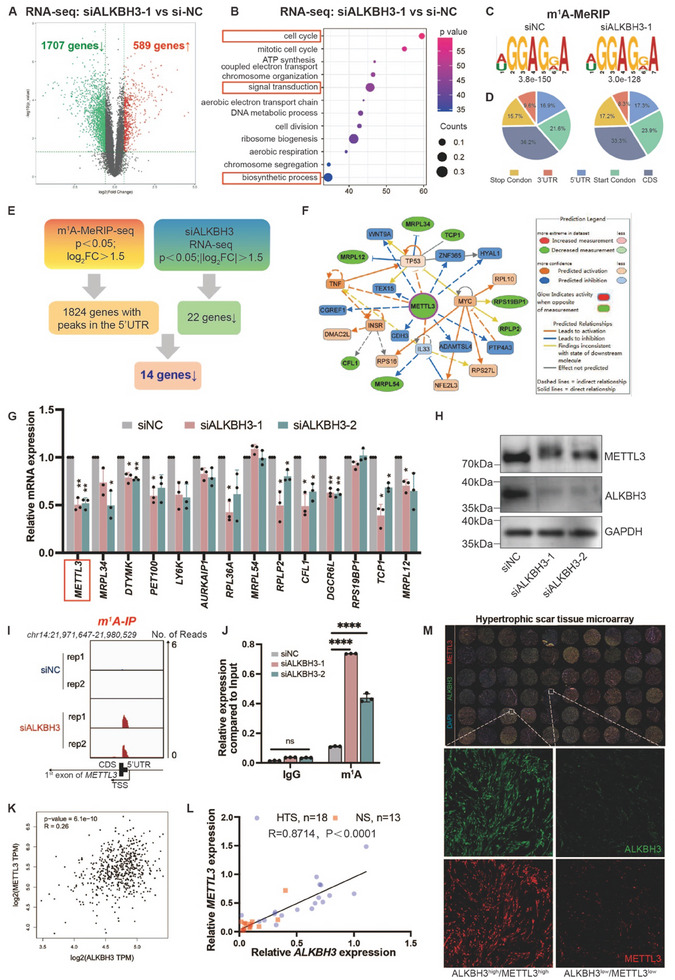
Characterization of m^1^A‒modified genes regulated by ALKBH3 in skin fibroblasts. A) Volcano plots showing ALKBH3‐regulated genes in wild‐type and ALKBH3‐deficient skin fibroblasts. B) GO enrichment map of ALKBH3‐regulated genes in wild‐type and ALKBH3‐deficient skin fibroblasts. C) m^1^A‐MeRIP‐seq data showing the top enriched motifs within m^1^A peaks identified in wild‐type and ALKBH3‐deficient skin fibroblasts. D) Pie charts showing the m^1^A peak distribution in different RNA regions (CDS, 5′ UTR, 3′ UTR, start codon, and stop codon) in wild‐type and ALKBH3‐deficient skin fibroblasts. E) Flowchart of the downstream analysis of m^1^A modifications. A total of 1824 genes with increased m^1^A peaks were identified in skin fibroblasts by m^1^A‐MeRIP‐seq. A total of 22 genes downregulated after ALKBH3 knockdown were identified by RNA‐seq; the cut‐off criteria used to define downregulated genes were a |log2FC| > 1.5 and *p* < 0.05. Finally, 14 candidate genes with both an increase in m^1^A peaks and a decrease in mRNA expression were identified as potential downstream targets of ALKBH3. F) Regulatory network of *METTL3* and 13 other candidate genes generated by IPA. Note that *DTYMK, PET100, LY6K, AURKAIP1, RPL36A* and *DGCR6L* are not shown because they had no pathway connections with *METTL3*. G) The expression of 14 candidate genes after ALKBH3 knockdown was measured using qRT‒PCR. The data are presented as the mean ± SD of triplicate experiments. Significance was determined by two‐way ANOVA, ^*^ *p* < 0.05; ^**^ *p* < 0.01. H) Western blot showing METTL3 expression relative to GAPDH in HDFs upon ALKBH3 knockdown. I) IGV tracks from the m^1^A‐meRIP‐seq analysis showing enrichment of m^1^A on *METTL3* transcription. J) Increased m^1^A modification in *METTL3* transcripts after ALKBH3 knockdown in HDFs, as assessed by gene‐specific m^1^A‐RIP‐qPCR assays. The experiments were performed in triplicate, and the relative expression of mRNA in each group was compared to the input and is shown as the mean ± SD. Significance was determined by unpaired two‐tailed Student's t test. ^****^ *p* < 0.0001. K) Correlation analysis of ALKBH3 expression and METTL3 expression in the GEPIA2 database. Significance was determined by *Pearson* correlation analysis (R = 0.26, *p* = 6.1e‐10). L) Correlation analysis between the expression of *ALKBH3* and *METTL3* in HTS (*n* = 18) and NS tissues (*n* = 13) (R = 0.8714, *p *< 0.0001). The relative expression levels were normalized to the GAPDH expression level. M) Immunofluorescence of ALKBH3 (green), METTL3 (red) and DAPI (blue) in the HTS tissue microarray. Scale bars: 500 µm. FC, fold change; IPA, Ingenuity Pathway Analysis, HTS, hypertrophic scar; NS, normal skin.

We then investigated the mechanism underlying the inhibitory effect of ALKBH3 silencing on skin fibrosis. After silencing ALKBH3 in HDFs, we performed multiomics analyses, including m^1^A‐MeRIP‐seq and RNA‐seq. As a result, silencing ALKBH3 led to notable alterations in the transcriptome, with 1707 genes downregulated and 589 upregulated (**Figure** **4**A). Since most of the differentially expressed genes (74.3% of the 2296 genes) were downregulated in ALKBH3‐deficient cells, our results are consistent with previous observations suggesting that ALKBH3‐induced m^1^A demethylation increases mRNA stability and consequently promotes gene expression.^[^ ^18^ ^]^ Moreover, the differentially expressed genes were associated with processes related to skin development and homeostasis, including process terms such as cell cycle, biosynthetic process, and signal transduction (Figure 4B). Additionally, the average numbers of m^1^A peaks detected in the m^1^A‐MeRIP‐seq libraries generated from control and ALKBH3‐deficient cells were 14 301 and 13 394, respectively (Figure 4C,D). These m^1^A peaks exhibited significant enrichment in 5′ UTRs, particularly near start codons (Figure S8A, Supporting Information). Notably, the differentially expressed m^1^A‐modified genes were associated with multiple fibrosis‐related pathways, including the MAPK, Hippo, and Wnt signaling pathways (Figure S8B,C, Supporting Information). These findings underscore the regulatory role of ALKBH3 in skin fibrosis pathogenesis.

By integrated analysis of these omics data, we identified 14 candidate genes potentially linked to ALKBH3‐regulated m^1^A modification (Figure 4E). Subsequent Ingenuity Pathway Analysis (IPA) of these 14 genes revealed that *METTL3* was located in the core of the candidate gene matrix (Figure 4F). We further confirmed that ALKBH3 silencing reduced METTL3 expression at both the mRNA and protein levels in fibroblasts, with a corresponding increase in m^1^A levels (Figure 4G–J; Figure S9A,B, Supporting Information). Similar findings were observed in *Alkbh3^−/−^* mice (Figure S9C,D, Supporting Information). Additionally, a significant positive correlation between ALKBH3 and METTL3 expression was found in skin samples, both in the GEPIA2 public database (R = 0.26, p value = 6.1e‐10) (Figure 4K) and in a clinical cohort via qRT‒PCR (R = 0.8714, *p* < 0.0001) (Figure 4L). This correlation was further validated by HTS tissue microarray staining, which revealed either a high/high or low/low ALKBH3/METTL3 expression pattern (R = 0.7428, *p* < 0.0001) (Figure 4M; Figure S9E, Supporting Information).

Notably, METTL3‐mediated m^6^A methylation has been identified as a critical factor in fibrotic processes such as cardiovascular and pulmonary fibrosis.^[^ ^19^, ^20^ ^]^ By analyzing clinical samples, we found that METTL3 and m^6^A methylation play a crucial role in the occurrence of pathological skin fibrosis (Figure S10, Supporting Information). We utilized two distinct siRNAs to silence METTL3 expression in HDFs, which resulted in a significant reduction in the m^6^A level (Figure S11, Supporting Information). Furthermore, the inhibition of METTL3 led to a decrease in the collagen deposition function and the growth capacity of fibroblasts, indicating a profibrotic role of METTL3 in pathological skin fibrosis (Figures S11 and S12, Supporting Information).

These findings corroborate the profibrotic actions of ALKBH3 in pathological skin fibrosis. Taken together, this suggests that METTL3, an m^1^A‐modified gene, is potentially regulated by ALKBH3.

### 2.5 The ALKBH3‐METTL3 Axis is Critical in Skin Fibrosis Pathogenesis

**Figure 5:**
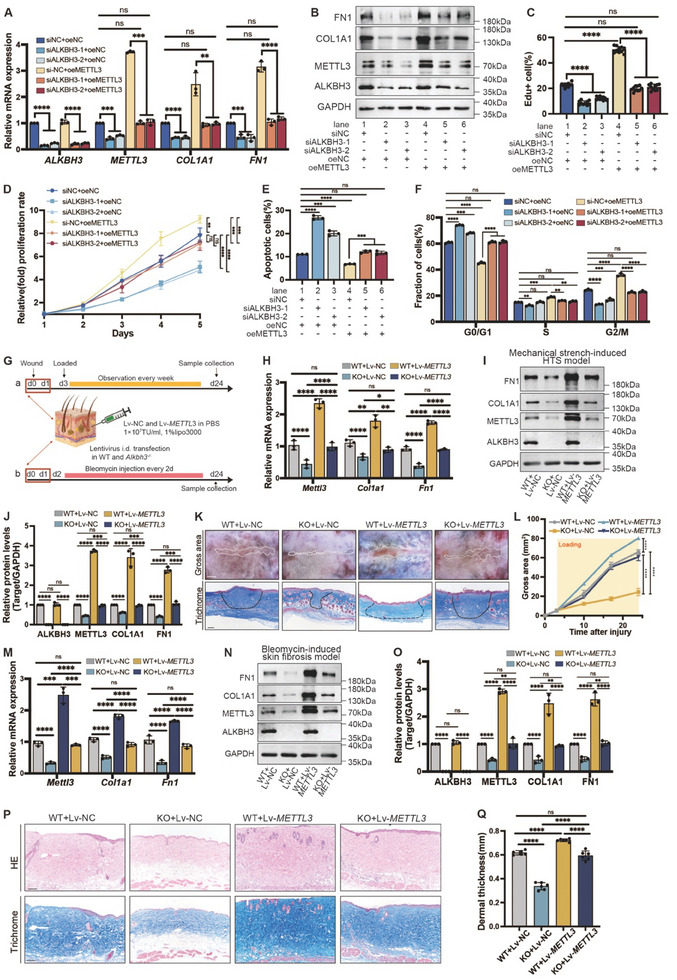
Exogenous overexpression of METTL3 counteracts the antifibrotic effects of ALKBH3 knockdown in vitro and in vivo. A) qRT‒PCR data analysis of *ALKBH3, METTL3, COL1A1*, and *FN1* mRNA levels in HDFs transfected with the ALKBH3 or NC siRNA and transduced with the METTL3 or NC lentiviral vector for 48 h. Data are mean ± SD of triplicate. Significance by unpaired two‐tailed Student's t test. ^***^ *p *< 0.001, ^****^ *p *< 0.0001. B) Western blot of ALKBH3, METTL3, COL1A1 and FN1 protein levels relative to GAPDH in ALKBH3‐deficient HDFs with METTL3 overexpression. C) Statistical analysis of the EdU incorporation assay results. Data are mean± SD of triplicate (10 fields analysed). Significance by unpaired two‐tailed Student's t test. ^****^ *p *< 0.0001 (D) CCK‐8 assay evaluated the proliferation of ALKBH3‐deficient HDFs with METTL3 overexpression. Data are mean± SD of triplicate. Significance by unpaired two‐tailed Student's t test. ^***^ *p *< 0.001, ^****^ *p *< 0.0001. E) Statistical analysis of the flow cytometric apoptosis assay in ALKBH3‐knockdown HDFs with METTL3 overexpression. Data are mean± SD of triplicate. Significance by unpaired two‐tailed Student's t test. ** *p*< 0.01, *** *p*< 0.001, ****P < 0.0001. (F) Statistical analysis of the cell cycle assay in ALKBH3‐knockdown HDFs with METTL3 overexpression. Data are mean± SD of triplicate. Significance by unpaired two‐tailed Student's t test. ^**^ *p *< 0.01, ^***^ *p *< 0.001, ^****^ *P* < 0.0001. G)Schematic diagram of intradermal injection of Lv‐*METTL3* and Lv‐NC (1 × 10^7^ TU mL^−1^, 1% Lipofectamine 3000) into WT and *Alkbh3* ^‒/‒^ mice 2 weeks before model establishment. a. Mechanical stretch‐induced HTS model. b. Bleomycin‐induced skin fibrosis model. Mice were sacrificed on day 24 post‐wounding (PWD24). H) qRT‒PCR of *Alkbh3*, *Mettl3, Col1a1*, and *Fn1* expression in WT and *Alkbh3* ^−/−^ mice injected with Lv‐*METTL* *3* or Lv‐NC in the mechanical stretch‐induced HTS model. Data are mean± SD of triplicate. Significance by unpaired two‐tailed Student's t test. ^*^ *p* < 0.05, ^**^ *p* < 0.01, ^****^ *p* < 0.0001. I) Western blot of ALKBH3, METTL3, COL1A1, and FN1 relative to GAPDH in the 4 groups of the mechanical stretch‐induced HTS model. J) Densitometric analysis of METTL3, COL1A1, and FN1 protein levels relative to GAPDH in the mechanical stretch‐induced HTS model. Data are mean± SD of triplicate. Significance by unpaired two‐tailed Student's t test. ^***^ *p* < 0.001, ^****^ *p* < 0.0001. K) Representative gross appearance (scale bar: 500 µm) and Masson staining (scale bar: 100 µm) of scar tissue in the mechanical stretch‐induced HTS model. L) Quantification of the scar area on PWD3, PWD10, PWD17, and PWD24 in the mechanical stretch‐induced HTS model. ^****^ *p* < 0.0001. M) qRT‒PCR of *Alkbh3, Mettl3, Col1a1*, and *Fn1* in WT and *Alkbh3* ^−/−^ mice injected with Lv‐*METTL* *3* or Lv‐NC in the bleomycin‐induced skin fibrosis model. Data are mean± SD of triplicate. Significance by unpaired two‐tailed Student's t test. ^***^ *p* < 0.001, ^****^ *p* < 0.0001. N) Western blot of ALKBH3, METTL3, COL1A1, and FN1 relative to GAPDH in the bleomycin‐induced skin fibrosis model. (O) Densitometric analysis of METTL3, COL1A1, and FN1 protein levels relative to GAPDH in the bleomycin‐induced skin fibrosis model. Data are mean± SD of triplicate. Significance by unpaired two‐tailed Student's t test. ^**^ *p* < 0.01, ^****^ *p* < 0.0001. P) HE and Masson staining of tissues in the bleomycin‐induced skin fibrosis model (scale bar: 100 µm). Q) Statistical analysis of dermal thickness in the bleomycin‐induced skin fibrosis model. Significance by unpaired two‐tailed Student's t test. ^****^ *p* < 0.0001. HTS, hypertrophic scar; HDF, hypertrophic scar derived fibroblast.

To verify the relationship between ALKBH3 and METTL3, we altered METTL3 expression after ALKBH3 inhibition in HDFs by exogenously overexpressing METTL3. As expected, the expression of METTL3 was significantly increased at both the mRNA (**Figure** **5**A) and protein levels (Figure 5B; Figure S13A, Supporting Information). Furthermore, the reintroduction of METTL3 effectively restored the endogenous METTL3 expression that had been reduced by ALKBH3 knockdown (Figure 5A,B; Figure S13A, Supporting Information). Notably, exogenous METTL3 overexpression also mitigated the inhibitory effects of ALKBH3 depletion on collagen deposition (Figure 5A,B; Figure S13A, Supporting Information) and significantly rescued the proliferative capacity of fibroblasts with ALKBH3 knockdown (Figure 5C–F; Figure S13B–D, Supporting Information). To validate these findings in vivo, we utilized *Alkbh3^−/−^* mice and overexpressed METTL3 with a lentiviral vector (Lv‐*METTL3*) following a previously established lentiviral transfection strategy (Figure 5G).^[^ ^21^ ^]^ In the mechanical stretch‐induced HTS model, METTL3 overexpression (Figure 5H–J) reversed the effects of ALKBH3 knockout, including reduced scar area and collagen deposition (Figure 5H–L). A similar reversal of dermal thickness and collagen deposition was observed in the bleomycin‐induced fibrosis model (Figure 5M–Q).

**Figure 6:**
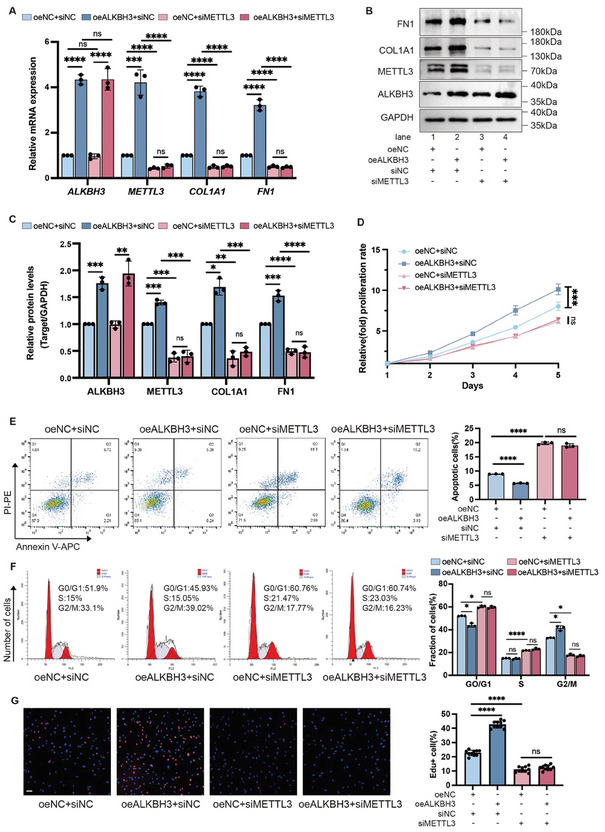
Inhibition of METTL3 suppressed the pro‐fibrotic effect of ALKBH3 in vitro. A) qRT‒PCR data showing the mRNA expression of *ALKBH3*, *METTL3*, *COL1A1*, and *FN1* in ALKBH3‐overexpressing HDFs with METTL3 knockdown. The data are presented as the mean± SD of triplicate experiments. Significance was determined by unpaired two‐tailed Student's t test. ^***^ *p *< 0.001, ^****^ *p *< 0.0001. B) Western blot showing ALKBH3, METTL3, COL1A1, and FN1 expression relative to GAPDH in ALKBH3‐overexpressing HDFs with METTL3 knockdown. C) Densitometric analysis of ALKBH3, METTL3, COL1A1 and FN1 protein levels relative to GAPDH in ALKBH3‐overexpressing HDFs with METTL3 knockdown. The data are presented as the mean ± SD of triplicate experiments. Significance was determined by unpaired two‐tailed Student's t test. ^*^ *p *< 0.05, ^**^ *p *< 0.01, ^***^ *p *< 0.001, ^****^ *p *< 0.0001. D) A CCK‐8 assay was used to evaluate the proliferation of ALKBH3‐overexpressing HDFs with METTL3 knockdown. The data are presented as the mean ± SD of triplicate experiments. Significance was determined by unpaired two‐tailed Student's t test. ^***^ *p *< 0.001. E) Apoptosis in ALKBH3‐overexpressing HDFs with METTL3 knockdown was analyzed by flow cytometry. All of the experiments were performed in triplicate. Significance was determined by unpaired two‐tailed Student's t test. ^****^ *p* < 0.0001. F) The cell cycle distribution of ALKBH3‐overexpressing HDFs with METTL3 knockdown was analyzed by flow cytometry. All of the experiments were performed in triplicate. Significance was determined by unpaired two‐tailed Student's t test. ^*^ *p *< 0.05, ^****^ *p *< 0.0001. G) The EdU (red) incorporation assay showed the effect of exogenous METTL3 overexpression on the proliferation of ALKBH3‐overexpressing HDFs with METTL3 knockdown. Scale bar: ALKBH 50 µm. The data are presented as the mean ± SD of triplicate experiments, and ten random fields were included in the analysis. Significance was determined by unpaired two‐tailed Student's t test. ^****^ *p *< 0.0001. HDF, hypertrophic scar derived fibroblast.

Additionally, to further confirm the dependency of ALKBH3's pro‐fibrotic effects on METTL3, we conducted experiments in which ALKBH3 was overexpressed while METTL3 was simultaneously knocked down in HDFs. As expected, the simultaneous ALKBH3‐overexpression and METTL3‐silenced group showed comparable METTL3 expression, compared to NC group (**Figure** **6**A–C; lanes 1 and 4). Consistently, the profibrotic effects of ALKBH3‐overexpression, including collagen deposition (Figure 6A–C) and proliferative capacity (Figure 6D–G), were diminished upon METTL3 deprivation, supporting the hypothesis that ALKBH3 exerts its profibrotic effects through METTL3‐dependent pathways.

### 2.6 ALKBH3 Regulates the Expression of METTL3 in a YTHDF2‐Dependent Manner

**Figure 7:**
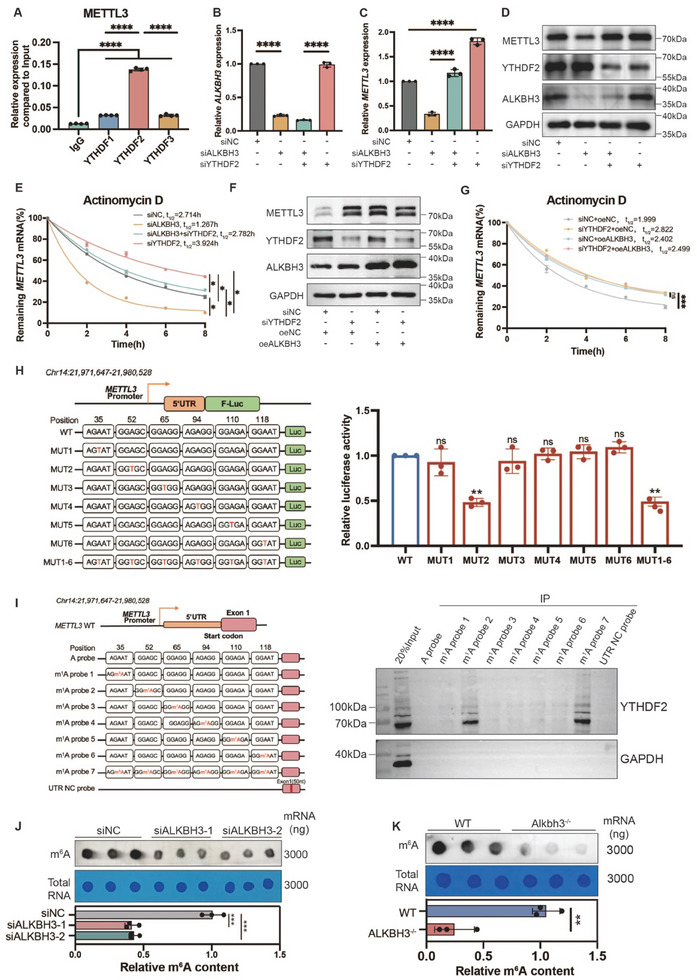
ALKBH3 regulates the expression of METTL3 in a *YTHDF2*‐dependent manner. A) RIP‒qPCR analysis revealed the enrichment of YTHDF1, YTHDF2, and YTHDF3 on the *METTL3* transcript. The data are presented as the mean ± SD of quadruplicate experiments. Significance was determined by unpaired two‐tailed Student's t test. ^****^ *p* < 0.0001. B) *ALKBH3* expression in HDFs upon ALKBH3 and YTHDF2 knockdown was measured by qRT‒PCR. The data are presented as the mean± SD of triplicate experiments. Significance was determined by unpaired two‐tailed Student's t test. ^****^ *p* < 0.0001. C) *METTL3* expression in HDFs upon ALKBH3 and YTHDF2 knockdown was measured by qRT‒PCR. The data are presented as the mean± SD of triplicate experiments. Significance was determined by unpaired two‐tailed Student's t test. ^****^ *p* < 0.0001. D) Western blot showing ALKBH3, YTHDF2, and METTL3 expression relative to GAPDH in HDFs upon ALKBH3 and YTHDF2 knockdown. E) Half‐life of *METTL3* in HDFs with ALKBH3 and YTHDF2 knockdown after treatment with actinomycin D (5 µg mL^−1^) for 0, 2, 4, 6, or 8 h. The mRNA expression of *METTL3* was measured by qRT‒PCR. The experiments were performed in triplicate. F) Western blot showing YTHDF2, ALKBH3, and METTL3 expression relative to GAPDH in ALKBH3‐overexpressing HDFs with YTHDF2 knockdown. G) Half‐life of METTL3 in ALKBH3‐overexpressing HDFs with YTHDF2 knockdown after treatment with actinomycin D (5 µg mL^−1^) for 0, 2, 4, 6, or 8 h. The experiments were performed in triplicate. H) The luciferase reporter gene assay demonstrated the relative luciferase activity of the wild‐type and seven mutant *METTL3* 5′UTR reporter vectors. The data are presented as the means ± SD of experimental triplicates. Significance was determined by unpaired two‐tailed Student's t test. ^**^ *p* < 0.01. I) Diagram showing the RNA probes used for RNA pull–down assays. RNA pull–down of the endogenous YTHDF2 protein from cell extracts using *METTL3* RNA probes with or without m^1^A modifications. J) Dot blot showing the m^6^A signal relative to the methylene blue signal in HDFs upon ALKBH3 knockdown. The data are presented as the mean ± SD of triplicate experiments. Significance was determined by unpaired two‐tailed Student's t test. ^***^ *p* < 0.001. K) Dot blot showing the m^6^A signal relative to the methylene blue signal in the skin of WT and *Alkbh3^‒/‒^* mice. The data are presented as the mean ± SD of triplicate experiments. Significance was determined by unpaired two‐tailed Student's t test. ^**^ *p* < 0.01.

We then explored the epigenetic mechanisms underlying the ALKBH3‐mediated m^1^A modificationl of METTL3. Since previous studies have demonstrated that YTHDF proteins are responsible for the recognition of m^1^A, we first tested the binding status of YTHDF1, YTHDF2, and YTHDF3 to *METTL3* mRNA. RNA immunoprecipitation (RIP) demonstrated that YTHDF2 specifically recognized *METTL3* mRNA. However, YTHDF1 and YTHDF3 exhibited only limited interactions with *METTL3* mRNA (**Figure** **7**A). Moreover, YTHDF2 expression exhibited a strong positive correlation (*R* = 0.12, *p* value = 0.0041) with METTL3 expression in skin samples represented in the GEPIA2 public database (Figure S14A, Supporting Information), completely consistent with the hypothesis that YTHDF2 is necessary for the recognition of METTL3.

Since YTHDF2 is responsible for RNA degradation,^[^ ^22^ ^]^ we examined its role in *METTL3* mRNA stability. Intriguingly, knockdown of ALKBH3 decreased the RNA stability of *METTL3*; however, this effect was reversed by subsequent depletion of YTHDF2 (Figure 7B–E: Figure S14B, Supporting Information). Furthermore, we have performed overexpression of ALKBH3 combined with YTHDF2 knockdown and compared the results to the group with ALKBH3 overexpression alone (Figure 7F; Figure S14C,D, Supporting Information). Our findings indicate that YTHDF2, as an m^1^A reader protein, promotes *METTL3* degradation and therefore inhibits its expression. On the contrary, upon ALKBH3 overexpression, which removes the m^1^A methylation modification, YTHDF2 knockdown did not impact the mRNA stability of *METTL3* (Figure 7G). These loss‐of‐function and gain‐of‐functions results aggregate the fact that YTHDF2 promotes *METTL3* degradation by recognition of m^1^A methylation of *METTL3*.

We then determined the specific m^1^A modification site of *METTL3* mRNA. Previous literature has highlighted that m^1^A methylation sites are often enriched in the 5′ UTR and near the start codon region of mRNAs.^[^ ^11^ ^]^ In the 5′ UTR of *METTL3*, we identified six potential m^1^A sites based on m^1^A‐meRIP‐seq analysis [c.35A (AGAAT), c.52A (GGAGC), c.65A (GGAGG), c.94A (AGAGG), c.110A (GGAGA), and c.118A (GGAAT), identified in the m^1^A‐seq peak, and mutated each A to a T. These wild‐type and mutant 5′ UTR sequences were then cloned into the pmirGLO vector. The luciferase reporter assay revealed that the c.A52T mutation resulted in a significant decrease in luciferase activity, while mutations at other sites did not remarkably alter the luciferase signal (Figure 7H). Similarly, following previous protocol,^[^ ^23^ ^]^ we observed m^1^A‐probe2 (carrying c.52‐m^1^A methylation site) interacts with YTHDF2 in the RNA pulldown assay, which is identical with the m^1^A‐probe 7 (positive control group). However, other m^1^A probes and control probe showed limited signals (Figure 7I). Taken together, these results showed c.52‐m^1^A methylation site serves as the recognition site of YTHDF2, which is responsible for the RNA degradation of *METTL3*.

Notably, METTL3 is a well‐known m^6^A writer and a crucial component of the m^6^A methyltransferase complex.^[^ ^24^ ^]^ We found that the reduced expression of METTL3 in ALKBH3‐deficient cells and *Alkbh3^−/−^* mice resulted in decreased overall m^6^A levels (Figure 7J,K). Collectively, these data shedding light on a novel process of “RNA methylation crosstalk” between m^1^A and m^6^A methylation.

### 2.7 METTL3, Relying on YTHDF1, Controls the m^6^A Modification and Stabilization of *COL1A1* and *FN1* mRNAs

**Figure 8:**
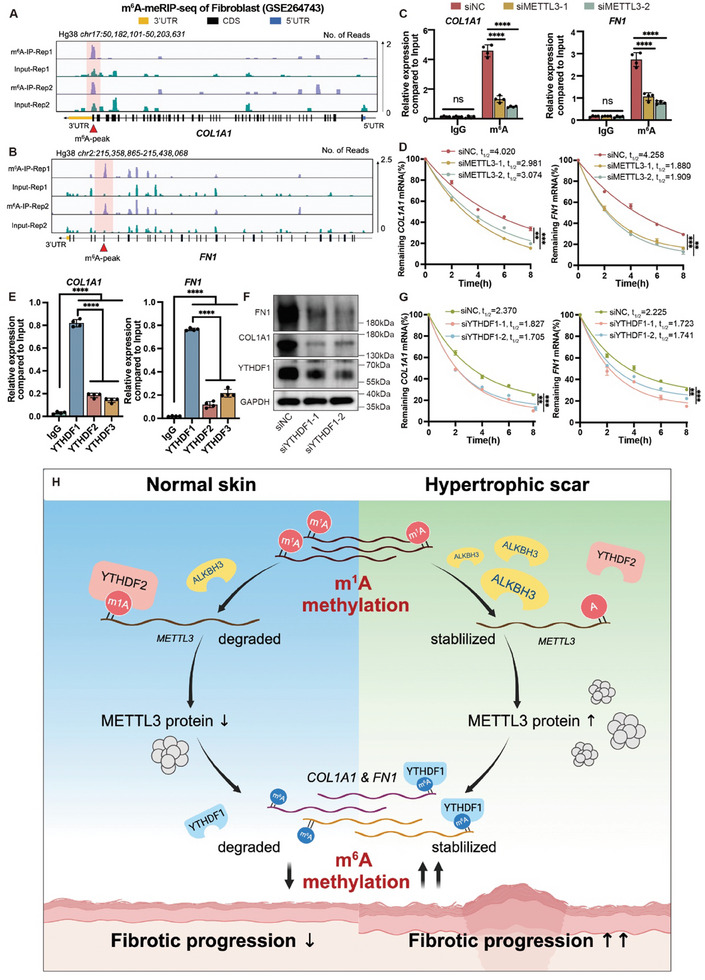
METTL3, relying on YTHDF1, controls the m^6^A modification and stabilization of *COL1A1* and *FN1 mRNAs*. A) IGV tracks from the m^6^A‐meRIP‐seq analysis showing enrichment of m^6^A on *COL1A1* transcription. B) IGV tracks from the m^6^A‐meRIP‐seq analysis showing enrichment of m^6^A on *FN1* transcription. C) Decreased m^6^A modification in *COL1A1* and *FN1* transcripts after METTL3 knockdown in HDFs, as assessed by gene‐specific m^6^A‐RIP‐qPCR assays. The experiments were performed in quadruplicate, and the relative expression of mRNA in each group was compared to the input and is shown as the mean ± SD. Significance was determined by unpaired two‐tailed Student's t test. ^****^ *p* < 0.0001. D) Half‐life of *COL1A1* and *FN1* in METTL3‐deficient HDFs treated with actinomycin (5 µg µL^−1^) for 0, 2, 4, 6, or 8 h. The mRNA expression was measured by qRT‒PCR. The experiments were performed in triplicate. E) RIP‒qPCR analysis revealed the enrichment of YTHDF1, YTHDF2, and YTHDF3 on the *COL1A1* and *FN1* transcript. The data are presented as the mean ± SD of quadruplicate experiments. Significance was determined by unpaired two‐tailed Student's t test. ^****^ *p* < 0.0001. (F) Western blot showing METTL3, YTHDF1, COL1A1 and FN1 expression relative to GAPDH in HDFs upon YTHDF1 knockdown. G) Half‐life of *COL1A1* and *FN1* in YTHDF1‐deficient HDFs treated with actinomycin (5 µg µL^−1^) for 0, 2, 4, 6, or 8 h. The experiments were performed in triplicate. H) Schematic diagram showing “RNA methylation crosstalk” between m^1^A and m^6^A methylation. In pathological skin fibrosis, ALKBH3 increases METTL3 expression by removing m^1^A in a YTHDF2‐dependent manner. The elevated METTL3 levels further stabilize *COL1A1* and *FN1* mRNAs via YTHDF1‐mediated m^6^A modification, thereby promoting the progression of fibrosis.

To further investigate the downstream targets of METTL3, we performed m^6^A‐MeRIP‐seq analysis on HDFs. The MeRIP‐seq identified ≈9792 m^6^A peaks per sample. These m^6^A peaks were predominantly enriched in the 3′UTRs, particularly near the stop codon. Notably, significant m^6^A modification peaks were observed in the *COL1A1* and *FN1* genes, suggesting that these genes might be regulated by METTL3 (**Figure** **8**A,B). m^6^A RIP experiments further confirmed that the m^6^A modification levels of *COL1A1* and *FN1* were markedly reduced upon METTL3 knockdown (Figure 8C). Additionally, m^1^A RIP analysis of ALKBH3‐silenced HDFs revealed no significant changes in m^1^A modification levels, indicating that *COL1A1* and *FN1* expression is regulated through m^6^A rather than m^1^A (Figure S15, Supporting Information). These findings provide further support for the hypothesis that *COL1A1* and *FN1* are targets of METTL3.

We then explored the detailed mechanism underlying the m^6^A regulation of *COL1A1* and *FN1* by METTL3. Since m^6^A modifications have been revealed to play vital roles in the maintenance of RNA stability, we first tested whether METTL3 regulates the RNA stability of *COL1A1* and *FN1*. As a result, we found that the RNA stability was dramatically decreased in METTL3‐deficient cells (Figure 8D), consistent with previous observations that METTL3 enhances the expression of COL1A1 and FN1. Importantly, YTHDF family members are also the major components responsible for recognizing m^6^A‐modified transcripts,^[^ ^25^ ^]^ we attempted to identify the potential reader protein. First, RIP analysis revealed that both *COL1A1* and *FN1* showed a strong interaction with YTHDF1; while very limited signals were captured in the anti‐YTHDF2/YTHDF3 groups (Figure 8E). To confirm the role of YTHDF1 in regulating *COL1A1* and *FN1*, we silenced YTHDF1 with two individual siRNAs and measured the expression levels of *COL1A1* and *FN1*. Notably, YTHDF1 silencing resulted in a significant reduction in both mRNA expression levels and protein abundance (Figure 8F; Figure S16, Supporting Information), accompanied by decreased RNA stability (Figure 8G), while protein stability remained unaltered (Figure S17, Supporting Information). These findings indicated that YTHDF1 acts as a nucleus m^6^A reader, stabilizing m^6^A‐modified mRNA, thereby highlighting the crucial role of m^6^A in collagen regulation.

However, m^6^A ‐MeRIP‐seq analysis revealed that another critical collagen gene, *COL3A1*, did not exhibit significant m^6^A modification peaks, suggesting that *COL3A1* expression is not influenced by METTL3‐mediated m^6^A methylation (Figure S18, Supporting Information). It is well known that the imbalance in the COL1A1/COL3A1 ratio is a key factor in the formation of pathological scars, typically characterized by excessive deposition of COL1A1 and a relative reduction in COL3A1. Therefore, the high expression of METTL3 in pathological scars, through YTHDF1‐dependent m^6^A methylation, enhances the stability of *COL1A1* and *FN1* mRNAs, exacerbating the imbalance in the COL1A1/COL3A1 ratio, ultimately affecting the structure and function of pathological skin fibrosis.

## 3 Discussion

In this study, we showed the crucial role of m^1^A‐m^6^A crosstalk in pathological skin fibrosis (Figure 8H). RNA modification is a crucial aspect of epigenetics and plays an important role in the mechanisms governing gene expression and cell fate determination. Recently, attention has been focused on the epigenetic regulatory network. For instance, diverse histone deacetylase (HDAC) inhibitors exert anticancer effects by modulating and orchestrating m^6^A modifications, revealing a “histone‐RNA crosstalk” process in ocular melanoma.^[^ ^26^ ^]^ Several studies have reported interactions between m^6^A and DNA methylation,^[^ ^27^, ^28^ ^]^ such as the interaction between YTHDC2 and TET1, which is crucial for transposon activity and the fate of human embryonic stem cells (hESCs).^[^ ^27^ ^]^ Here, we propose the existence of an RNA modification network for the first time, uncovering the synergistic role of m^1^A and m^6^A modification‐related proteins in promoting pathological skin fibrosis, thereby broadening our current understanding of RNA methylation.

Previously, research on RNA methylation modifications has predominantly focused on their oncogenic role in cancer, including impacts on cell proliferation and apoptosis, invasion and metastasis, and metabolic reprogramming. For instance, ALKBH3‐mediated SP100 demethylation has been implicated in tumorigenesis in ocular melanoma, as determined by both in vitro and in vivo experiments.^[^ ^13^ ^]^ Moreover, few studies have mentioned that METTL3, a canonical writer protein for m^6^A methylation, plays a pathogenic role in other fibrotic diseases. Notably, METTL3‐mediated m^6^A methylation promotes liver fibrosis by enhancing the secretion of TGF‐β.^[^ ^29^ ^]^ Additionally, the highly specific small‐molecule inhibitor of METTL3, STM2457 (Chemicals, DC53045, Shanghai, China), has been shown to attenuate kidney fibrosis in vivo.^[^ ^30^ ^]^ However, the relationship between RNA modifications and skin fibrosis remains unclear. In this study, we found that ALKBH3‐mediated m^1^A demethylation of METTL3 transcription affects the m^6^A methylation levels of collagen, leading to the development of skin fibrosis. This finding is the first to elucidate the critical roles of two types of methylation modifications in skin fibrotic diseases, thereby expanding our current understanding of methylation modifications.

We anticipate that targeting the ALKBH3‐METTL3 axis for the treatment of pathological skin fibrosis could be applied clinically in the near future. Although both ALKBH3 and METTL3 contribute to fibrosis, it is likely that ALKBH3 represents a more suitable clinical target for intervention. First, ALKBH3 functions as an upstream regulator of METTL3 and plays a central role in the regulation of skin fibrosis. Second, literature reports that METTL3 serves as an important regulator for immune homeostasis,^[^ ^31^, ^32^ ^]^ pro‐angiogenic functions,^[^ ^33^, ^34^ ^]^ wound healing process,^[^ ^35^, ^36^ ^]^ indicating that targeting METTL3 may result in various side effects.

To further advance clinical translation, we explored the clinical relevance of our findings and showed that treatment with ASO‐Alkbh3 effectively reduced fibrosis severity in a skin fibrosis model. ASOs can precisely modulate the transcript levels of both precursor mRNAs (pre‐mRNAs) and mature mRNAs, demonstrating notable target specificity. This capability could broaden the therapeutic landscape for a spectrum of diseases previously considered untreatable. For example, eteplirsen, which targets exon 51 of dystrophin pre‐mRNA, restores the translational reading frame and is approved for Duchenne muscular dystrophy (DMD) treatment.^[^ ^37^ ^]^ Additionally, nusinersen, an ASO drug targeting Survival motor neuron‐2 (SMN‐2) mRNA has been approved for the treatment of spinal muscular atrophy (SMA).^[^ ^38^ ^]^ Notably, the current treatments for skin fibrosis are limited and often ineffective, making ALKBH3‐targeted therapy a valuable therapeutic approach. However, the clinical application of ASOs has certain limitations: ASOs possess a high negative charge, are susceptible to enzymatic degradation and are rapidly cleared from the circulatory system.^[^ ^39^ ^]^ To address these challenges, researchers have explored various ASO delivery systems, including lipid nanoparticles, liposomes, polymer nanoparticles, and bioconjugates,^[^ ^40^, ^41^ ^]^ which hold promise for revolutionizing the treatment landscape of numerous diseases in the near future.

Despite the significant findings of this study, several limitations should be acknowledged, as they may affect the interpretation and generalizability of our results. First, due to the limited availability of clinical samples, HTSs were the only means by which we could validate the role of m^1^A in skin fibrosis. Nevertheless, in our study, we employed two animal models, namely the mechanical stretch‐induced HTS and bleomycin‐induced skin fibrosis models, both of which are classic representations of skin fibrosis diseases. Furthermore, while our study utilized ALKBH3 global knockout mice, we acknowledge the potential involvement of multiple cell types in the pathogenesis of skin fibrosis. Our comprehensive single‐cell analysis and functional validation studies consistently demonstrated that fibroblasts serve as the primary effector cells mediating ALKBH3‐dependent fibrotic processes. However, to further substantiate these findings and elucidate potential contributions from other cellular components, future studies employing conditional knockout models will be essential. This will provide more robust and specific evidence regarding the cell‐type‐specific role of ALKBH3 in skin fibrosis. Additionally, while ALKBH3 is known to exhibit diverse functions across various RNA species, including tRNAs, rRNAs, mRNAs, and mitochondrial RNAs,^[^ ^8^, ^9^ ^]^ its role in the m^1^A demethylation of tRNAs/rRNAs in the context of skin fibrosis remains unclear and warrants further investigation.

In summary, this study is the first to reveal the existence of “RNA methylation crosstalk” mediated by m^1^A and m^6^A, providing new insights into RNA modifications. Additionally, our findings reveal a novel fibrosis mechanism where ALKBH3‐mediated m^1^A demethylation enhances METTL3 expression, which in turn promotes fibrosis by elevating m^6^A levels of *COL1A1* and *FN1* transcripts. This insight offers a new avenue for targeted therapeutic intervention.

## 4 Experimental Section

### Patients and Tissue Specimens

Hypertrophic scar and normal skin samples were obtained from patients who underwent surgery at the Department of Plastic and Reconstructive Surgery at Shanghai Ninth People's Hospital, Shanghai Jiao Tong University School of Medicine. Written informed consent was obtained before sample collection in accordance with the Declaration of Helsinki and with approval from the Human Research Ethics Committee of Shanghai Jiao Tong University School of Medicine (patient information is summarized in Table S2, Supporting Information). The ethics permit number for the use of clinical samples collected during surgery was 2018‐129‐T107.

### Cell Culture and Treatment

The isolation and culture of fibroblasts from human HTS and NS tissue were conducted as previously described.^[^ ^42^ ^]^ Briefly, surgical specimens were dissected into 5 mm×5 mm pieces and incubated in 0.3% dispase II (0.3 g mL^−1^; Gibco, 17 105 041) at 4 °C for 12 h. Then, the epidermis was removed, and the dermis was minced and incubated in collagenase NB4 (3 mg mL^−1^; Nordmark, S1745401) at 37 °C for 4 h to isolate dermal fibroblasts. The EC and SMC cell line were purchased from the American Type Culture Collection (Manassas, VA, USA). Fibroblasts and ECs were cultured in DMEM (Gibco, USA) supplemented with 10% foetal bovine serum (FBS; Gibco, USA) and 1% penicillin‒streptomycin (Gibco, USA) at 37 °C in a humidified atmosphere with 5% CO_2._ Notably, the primary fibroblasts could be passaged up to 6 passages. SMCs were cultured in Smooth Muscle Cell Medium (SMCGS, Cat #1152, ScienCell) supplemented with 10 mL of fetal bovine serum (FBS, Cat. No. 0010, ScienCell), 5 mL of smooth muscle cell growth supplement (SMCGS, Cat. No. 1152, ScienCell), and 5 mL of penicillin/streptomycin solution (P/S, Cat. No. 0503, ScienCell) at 37 °C in a humidified atmosphere with 5% CO_2_.

### Dot Blot Assay

Total RNA was extracted using TRIzol Reagent (Invitrogen, USA), and 2 or 3 µg of RNA was spotted onto two separate nitrocellulose membranes (Millipore, INYC00010), one for total RNA detection and the other for m^1^A or m^6^A methylation analysis. For total RNA detection, the membrane was stained with 0.02% methylene blue for 30 min, followed by a brief wash with nuclease‐free water briefly before capture. For methylation analysis, the membrane was cross‐linked under ultraviolet (UV) light, blocked with 5% milk for 1 h at room temperature, and then incubated overnight at 4 °C with anti‐m^1^A antibody (ab208196, Abcam, USA) and anti‐m6A antibody (A19841, abclonal) at 4 °C overnight. Afterward, the membrane was incubated with horseradish peroxidase (HRP)‐conjugated anti‐rabbit IgG (#4412, CST, USA) for 1 h at room temperature (RT) and visualized using enhanced chemiluminescence (ECL) (Millipore, WBKLS0100).

### RNA Isolation and qRT–PCR

Total RNA was extracted from cultured cells using TRIzol Reagent (Invitrogen, USA), and cDNA was synthesized using PrimeScript RT Reagent Mix (Takara Bio, RR036A) according to the manufacturer's instructions. qRT‒PCR was performed on an ABI QuantStudio 6 Flex system using SYBR Premix (Takara, RR066A) according to the manufacturer's instructions. The sequences of the primers used are summarized in Table S3 (Supporting Information).

### Western Blot (WB) Analysis and IF Staining

WB analysis and IF staining were performed according to the protocol described in our previous study.^[^ ^43^ ^]^ The antibodies used for WB analysis and IF staining are listed in Table S4 (Supporting Information). The original WB images are available in Figures S19 and S20 (Supporting Information). Image‐Pro Plus 6.0 software was used for quantitative analysis.

### Plasmid Construction and RNA Interference

Knockdown of RNA expression in fibroblasts was achieved by transfection with siRNA sequences synthesized by Zorin Biotechnology Co., Ltd. (Shanghai, China). The sequences of the siRNAs used are listed in Table S5 (Supporting Information). The METTL3 and ALKBH3 overexpression cassettes were generated via PCR, inserted into MSCV and CMV vectors respectively, and confirmed by DNA sequencing. (the sequences used to construct the overexpression plasmid are summarized in Table S6, Supporting Information). Transfection of siRNAs and overexpression plasmids was performed using Lipofectamine 3000 transfection reagent (Invitrogen, L3000008) according to the manufacturer's instructions.

### Cell Proliferation Assays

CCK‐8 colorimetric assays were employed to assess cell proliferation. A total of 2000–3000 cells were seeded into 96‐well plates (Corning, USA) in 100 µL of medium. 3 h prior to detection, 10 µL of CCK‐8 solution (Dojindo, Japan) was added, and the samples were incubated at 37 °C. To measure the absorbance of the samples at 450 nm, a microplate reader (ELX800, BioTek, USA) was used.

### EdU Incorporation Assay

A total of 5000–6000 cells were seeded in 24‐well plates. After 24 h of incubation, cell proliferation was evaluated via the incorporation of EdU with an EdU Cell Proliferation Assay Kit (Invitrogen, Click‐iT EdU Imaging Kit, C10337). The steps were conducted according to the manufacturer's protocol. In brief, cells were incubated with 50 µm EdU for 3.5 h before fixation, permeabilization, and EdU staining. Then, the cell nuclei were stained with 4′,6‐diamidino‐2‐phenylindole (1 µg mL^−1^, DAPI; Sigma–Aldrich, D9542) for 20 min. The proportion of cells with EdU incorporation was determined with a Zeiss 710 laser scanning microscope (Thornwood, NY, USA).

### Apoptosis and Cell Cycle Assays

Apoptosis was assessed as in a previous study using a fluorescein isothiocyanate (FITC)‐Annexin V apoptosis kit (BD Biosciences, San Diego, CA) was described following the manufacturer's instructions.^[^ ^43^ ^]^ In brief, cells were washed twice with cold PBS and stained with FITC‐Annexin V and propidium iodide (PI) on ice for 5 min. For cell cycle analysis, cells were collected and fixed with 75% cold ethanol at 4 °C for 2 h. The cells were subsequently rinsed with PBS 3 times and stained sequentially with RNase A and PI (Cell Cycle Assay Kit, Dojindo, Japan) according to the manufacturer's instructions. The samples were subjected to flow cytometric analysis (BD LSRFortessa analyzer, BD Biosciences).

### Animals

Six‐ to eight‐week‐old C57BL/6 mice were purchased from Cyagen Biosciences, Inc. (Cyagen, Suzhou, China). *Alkbh3^−/−^* C57BL/6 mice were generated via the CRISPR/Cas9 system. Single‐guide RNAs (sgRNAs) were designed to target exons 3 to 5 of *Alkbh3* and were coinjected with Cas9 into the zygotes. The pups obtained were genotyped by PCR. After genotyping, the F0 mice were subjected to serial mating to generate homozygous mutant offspring.

### Animal Models

All the procedures for establishing the model were conducted in accordance with the Guide for the Care and Use of Laboratory Animals and were approved by the Committee of Animal Care and Use for Research and Education (CACURE) of Shanghai Jiao Tong University School of Medicine. The ethics permit number for the animal HTS model study was SH9H‐2021‐A215‐1.

The stretch‐induced HTS model was established according to the model developed by Aarabi et al.^[^ ^44^ ^]^ For the bleomycin‐induced skin fibrosis model, 100 µL of bleomycin solution (B8416, diluted to 1 unit mL^−1^; Sigma–Aldrich, St. Louis, MO) was injected intradermally into the dorsal skin at four symmetrically distributed injection sites every other day for 3 weeks.

### Lentiviral Packaging

A mixture of 3 µg of the indicated plasmid, 3 µg of the pMD2.D plasmid, and 6 µg of the PsPax plasmid was transfected into HEK239T cells with Lipofectamine 3000 (Invitrogen, L3000008) in Opti‐MEM I Reduced Serum Medium (Gibco, USA). The medium was replaced with fresh complete medium 6 h after transfection. Then, 48 and 72 h after transfection, the virus‐containing supernatant was collected, filtered through 0.45‐mm cellulose acetate filters, and concentrated with a Lenti‐X Concentrator (Takara Bio, USA).

### RNA‐seq and Data Analysis

Total RNA was extracted from fibroblasts using TRIzol reagent (Invitrogen, Carlsbad, CA, USA). Poly(T) oligo‐attached magnetic beads were used to enrich eukaryotic mRNA. After fragmentation, the mRNA was used to construct individual cDNA libraries. After cluster generation, the prepared libraries were sequenced on the Illumina NovaSeq 6000 platform. Gene expression levels were quantified as fragments per kilobase of exon model per million mapped reads (FPKM) values. The DESeq2 algorithm was used to identify differentially expressed genes, with a false discovery rate (FDR) < 0.05 and | log2(fold change) |≥ 1.5 as the thresholds.

Gene Ontology (GO) analysis of the designated genes was performed using the Database for Annotation, Visualization, and Integrated Discovery (DAVID) tool (http://david.abcc.ncifcrf.gov/). Fisher's exact test was used to identify the significant GO terms, and the FDR was used to correct the P values. GO terms with *P* < 0.05 were considered to be significantly enriched. Enrichment maps were created using Cytoscape 3.7.0, and bubble plots were constructed using GraphPad Prism 9.0 (GraphPad Software, Inc.). The correlation networks of METTL3 with other candidate genes was constructed using IPA software (Ingenuity Systems).

### MeRIP‐Seq and Data Analysis

MeRIP‐Seq referred to here as m^1^A‐MeRIP‐seq, was performed in accordance with published protocols with minor modifications.^[^ ^11^ ^]^ In brief, RNA was randomly fragmented into lengths of ≈200 nt with RNA fragmentation reagents, and protein A/G beads were coated with an anti‐m^1^A antibody (202 003, Synaptic Systems, Germany) by rotating at RT for 1 h. The RNA fragments were incubated with the beads at 4 °C for 4 h. Then, the captured RNA was eluted from the beads and isolated with TRIzol Reagent (Invitrogen, USA). Both the input sample and the m^1^A immunoprecipitated sample were used for library preparation with the NEBNext Ultra RNA Library Prep Kit (New England Biolabs, UK). The libraries were qualified with an Agilent 2100 bioanalyzer (Agilent, USA) and sequenced on the NovaSeq 6000 platform (Illumina, USA). The harvested paired‐end reads were subjected to quality control using Q30 and 3′ adaptor trimming by cutadapt software (v1.9.3) to remove low‐quality reads. Clean reads obtained from all libraries were aligned to the reference genome (hg19) using HISAT2 software (v2.0.4). Methylated sites on RNAs (peaks) were identified with MACS software, and differentially methylated sites were identified with diffReps. The peaks identified as overlapping with exons of mRNAs were chosen. In addition, GO and pathway enrichment analyses were performed on the differentially methylated protein‐coding genes. The MeRIP‒seq data were deposited in the Sequence Read Archive (SRA) database (PRJNA1114374). Sequencing was conducted by Kangcheng Biotech, Inc. (Shanghai, China).

### RNA‐Binding Protein Immunoprecipitation (RIP)‐qPCR

The m^1^A and RNA‐binding proteins were assessed by RIP experiments using an RNA immunoprecipitation kit (P0101, Geneseed, Shanghai, China) following the manufacturer's instructions. In brief, 1.0 × 10^7^ cells were treated with 1 mL of RIP lysis buffer. The resulting supernatants were divided into two fractions: 100 µL was kept as input, and 900 µL was incubated with specific antibody‐ or rabbit IgG‐conjugated protein A/G magnetic beads in IP buffer supplemented with RNase inhibitors at 4 °C overnight. The immunoprecipitated RNA was digested, purified, and further analyzed by qPCR. The primers and antibodies used for the RIP‐qPCR experiments are listed in Tables S3 and S4 (Supporting Information), respectively.

### RNA Stability

HDF cells were treated with actinomycin‐D (5 µg mL^−1^, HY‐17 559, MCE) for 0, 2, 4, 6, and 8 h, and the half‐life of the RNA was calculated by Prism GraphPad 9.0 (GraphPad Software, Inc.).

### Luciferase Reporter Assay

The DNA fragments of the METTL3‐5′UTR containing the wild‐type m^1^A motifs and mutant motifs (potential m^1^A was replaced by T) were synthesized and inserted upstream of the firefly luciferase of the pmirGLO vector. Cultured 293T cells were transfected with pmirGLO‐METTL3‐WT‐luc or pmirGLO‐METTL3‐MUT‐luc. Relative luciferase activity was evaluated 48 h after transfection by the Dual‐Luciferase Reporter Assay System (Promega, USA). The sequences are listed in Table S7 (Supporting Information).

### RNA Pull‐Down

RNA‒protein pull‒down assays were performed using a PureBinding RNA‒protein pull‒down kit (P0201, Geneseed, Shanghai, China) according to the manufacturer's instructions. Biotin‐labeled ssRNA probes were synthesized in vitro by Generay Biotechnology (Shanghai) Co., Ltd. (the sequences of the ssRNA probes are listed in Table S8, Supporting Information). Briefly, the cell pellets were resuspended and homogenized using 1 mL of standard lysis buffer. Five percent of each sample was used as input. Then, 100 pmol of RNA probes and 50 µL of magnetic beads were incubated with each sample at 4 °C for 1 h with rotation. The eluted protein and input samples were diluted using SDS‒PAGE loading buffer and analysed by WB.

### Protein Stability

Cycloheximide (100 µg mL^−1^, CHX; HY‐12320, MCE, USA) was added to HDF cells at predetermined intervals. Cells were harvested, and the protein stability of COL1A1 and FN1 was determined using western blotting.

### Statistical Analysis

GraphPad Prism 9.0 was used for statistical analysis. Quantitative data are presented as the mean ± SD values, and comparisons between two groups were performed by unpaired Student's t test. Correlations between two sets of data were assessed using simple linear regression analysis. If the variance among three or more groups was minimal, ANOVA followed by Dunnett's post hoc test or Tukey's post hoc test was used for multigroup comparisons. A *p* value <0.05 was considered to indicate statistical significance, and the number of asterisks denotes the level of statistical significance (^*^ *p* < 0.05, ^**^ *p* < 0.01, ^***^ *p* < 0.001, and ^****^ *p* < 0.0001).

## Conflict of Interest

The authors declare no conflict of interest.

## Author Contributions

L.T., S.G., R.X., and E.Y. contributed equally to this work. L.T. and S.G. designed and performed the experiments and drafted and revised the manuscript. R.X. and E.Y. designed and performed some of the experiments. S.L., X.H., X.L., and H.L. were responsible for the discussion and interpretation of the data. Y.Z. and T.Z. contributed to the revision and approval of the manuscript. All the authors reviewed and approved the final version of the manuscript.

## Supporting information



Supporting Information

## Data Availability

The data that support the findings of this study are available from the corresponding author upon reasonable request.
