# Supplementary material for: ALKBH3‐Mediated M1A Demethylation of METTL3 Endows Pathological Fibrosis:Interplay Between M1A and M6A RNA Methylation
Source: Adv Sci (Weinh). 2025 Feb 28;12(19):2417067. doi: 10.1002/advs.202417067 (PMC12097030; doi:10.1002/advs.202417067)
Supplement: Supplementary file 1 — Supporting Information [file ADVS-12-2417067-s001.pdf]

ADVANCED SCIENCE
Open Access

Supporting Information

for *Adv. Sci.*, DOI 10.1002/advs.202417067

ALKBH3-Mediated M[1]A Demethylation of METTL3 Endows Pathological Fibrosis:Interplay Between M[1]A and M[6]A RNA Methylation

Liying Tu, Shuchen Gu, Ruoqing Xu, En Yang, Xin Huang, Hsin Liang, Shenying Luo, Haizhou Li, Yixuan Zhao* and Tao Zan**

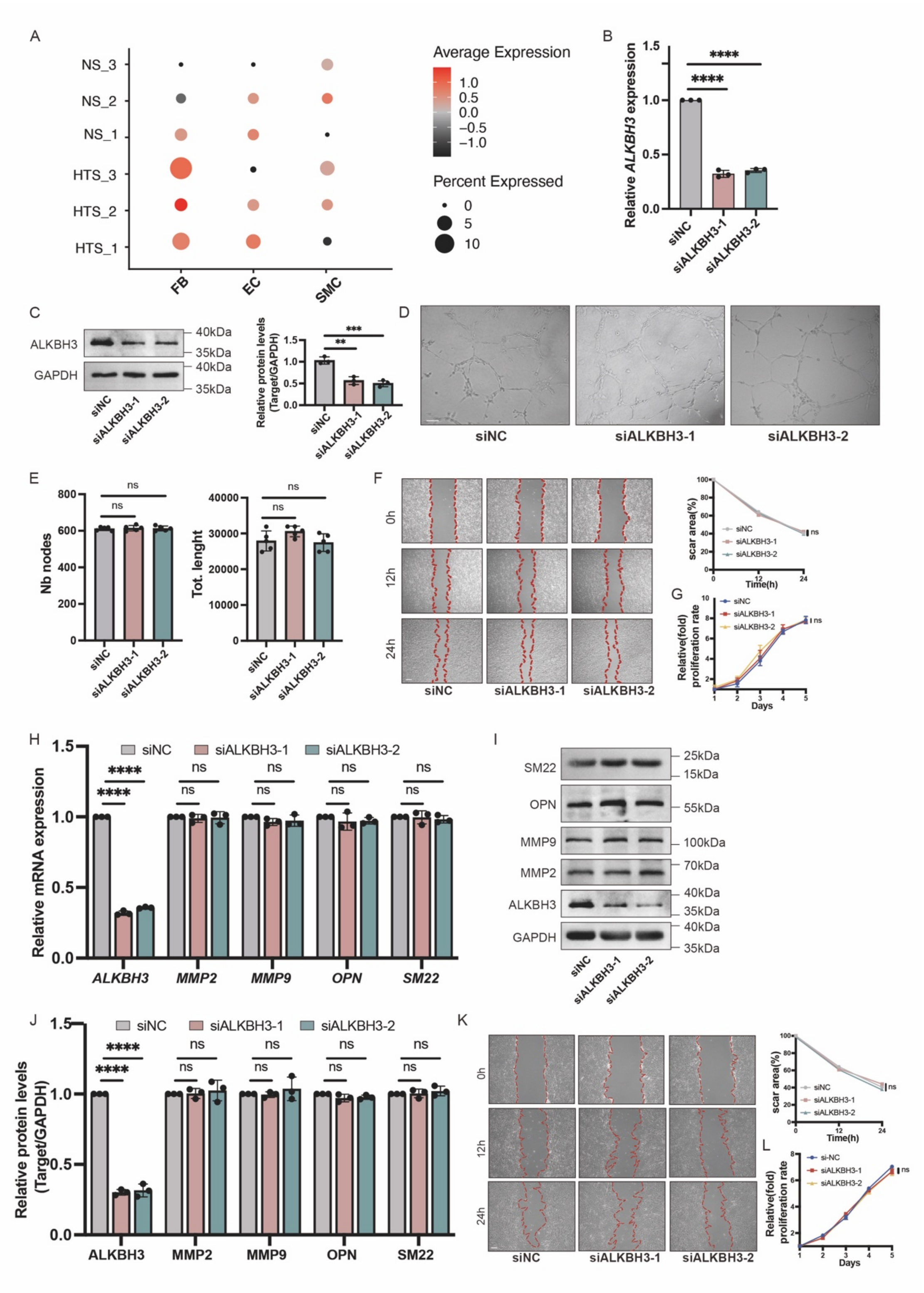

Figure S1. The functional effects of ALKBH3 on EC and SMC *in vitro*.

(A) Single-cell sequencing analysis revealed the expression of ALKBH3 in fibroblasts (FBs), endothelial cells (ECs), and smooth muscle cells (SMCs) in both hypertrophic scars (HTS) and normal skin (NS). (B) qRT–PCR data showing *ALKBH3* expression in

ECs upon ALKBH3 knockdown. The data are presented as the mean± SD of triplicate experiments. Significance was determined by unpaired two-tailed Student's *t* test. ****$p < 0.0001$. (C) Western blot and densitometric analysis showing ALKBH3 relative to GAPDH expression in ECs upon ALKBH3 knockdown. The data are presented as the mean ± SD of triplicate experiments. Significance was determined by unpaired two-tailed Student's t test. **$p < 0.01$, ****$p < 0.0001$. (D) The tube formation assays show the angiogenesis capacity of ECs upon ALKBH3 knockdown. (E) Densitometric analysis of the angiogenesis capacity of ECs upon ALKBH3 knockdown. (F) The long-term migratory ability of ECs was evaluated with wound healing assays. Scale bar: 100 μm. All of the experiments were performed in triplicate, and 3 random fields at each time point were included in the analysis. Significance was determined by unpaired two-tailed Student's *t* test. (G) A CCK-8 assay was used to evaluate the proliferation of ECs upon ALKBH3 knockdown. The data are presented as the mean ± SD of triplicate experiments. Significance was determined by unpaired two-tailed Student's *t* test. *$p < 0.05$. (H) qRT–PCR data showing *ALKBH3, MMP2, MMP9, OPN and SM22* expression in SMCs upon ALKBH3 knockdown. The data are presented as the mean ± SD of triplicate experiments. Significance was determined by unpaired two-tailed Student's *t* test. ****$p < 0.0001$. (I) Western blot showing ALKBH3, MMP2, MMP9, OPN and SM22 relative to GAPDH expression in SMCs upon ALKBH3 knockdown. (J) Densitometric analysis results showing the protein expression of ALKBH3, MMP2, MMP9, OPN and SM22 relative to that of GAPDH in SMCs upon ALKBH3 knockdown. The data are presented as the mean ± SD of triplicate experiments. Significance was determined by unpaired two-tailed Student's t test. ****$p < 0.0001$. （K）The long-term migratory ability of SMCs was evaluated with wound healing assays. Scale bar: 100 μm. All of the experiments were performed in triplicate, and 3 random fields at each time point were included in the analysis. Significance was determined by unpaired two-tailed Student's *t* test. (L) A CCK-8 assay was used to evaluate the proliferation of SMCs upon ALKBH3 knockdown. The data are presented as the mean ± SD of triplicate experiments. Significance was determined by unpaired two-tailed Student's *t* test. *$p < 0.05$.

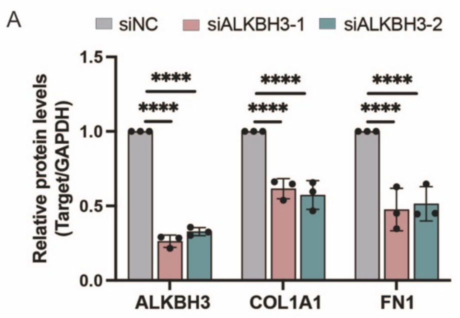

Figure S2. ALKBH3 knockdown attenuates the collagen deposition *in vitro*.

(A) Densitometric analysis results showing the protein expression of ALKBH3, COL1A1 and FN1 relative to that of GAPDH in HDFs upon ALKBH3 knockdown. The data are presented as the mean ± SD of triplicate experiments. Significance was determined by unpaired two-tailed Student's t test. **** $p < 0.0001$.

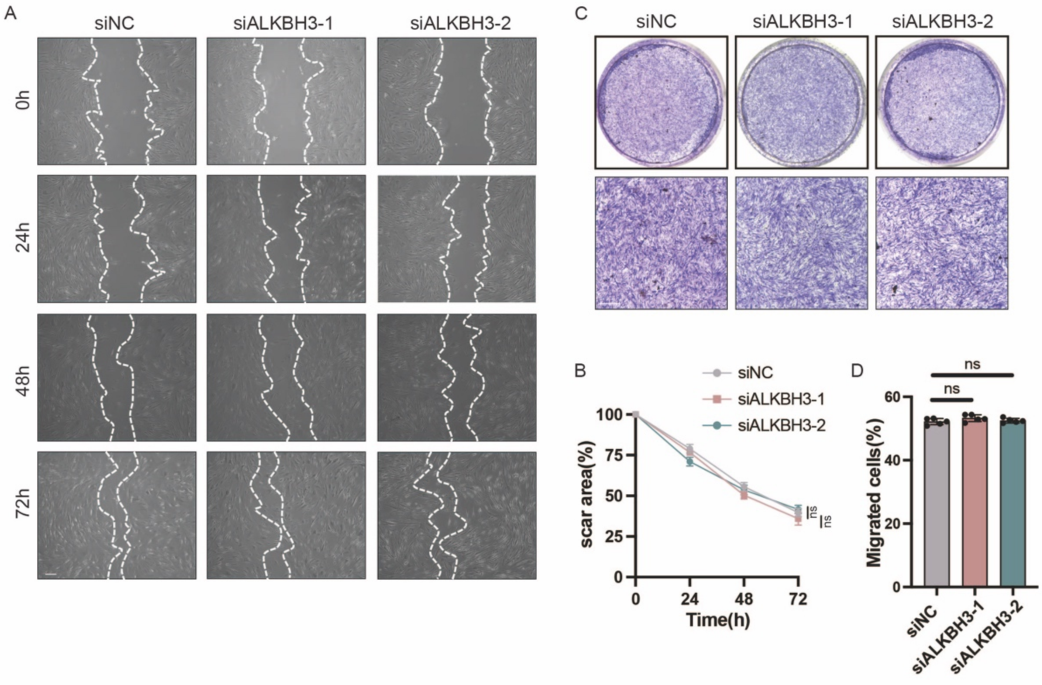

Figure S3. Inhibition of ALKBH3 had no effect on fibroblast migration *in vitro*.

(A) The long-term migratory ability of HDFs was evaluated with wound healing assays. Scale bar: 100 μm. (B) Statistical analysis of the wound healing assay results. All of the experiments were performed in triplicate, and 3 random fields at each time point were included in the analysis. Significance was determined by unpaired two-tailed Student's *t* test. ns, not significant. (C) The migration of HDFs after ALKBH3 knockdown was

analysed by a Transwell assay. Scale bar: 500 μm. ns, not significant. (D) Statistical analysis of the Transwell assay results. All of the experiments were performed in triplicate, and five random fields were included in the analysis. Significance was determined by unpaired two-tailed Student's *t* test. ns, not significant.

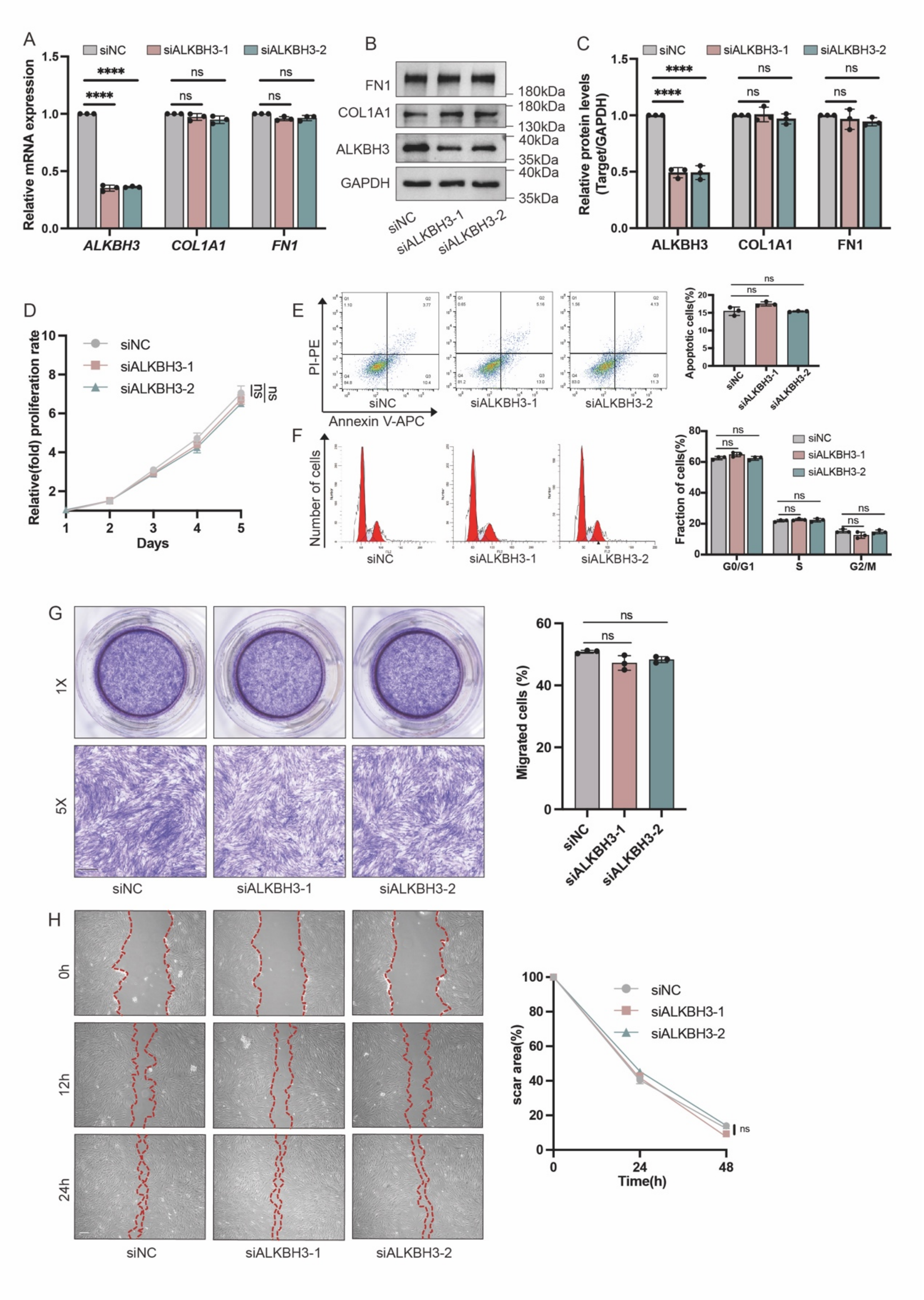

Figure S4. Inhibition of ALKBH3 in NDFs did not significantly affect fibroblast function *in vitro*.

(A) qRT–PCR data showing *ALKBH3, COL1A1 and FN1* expression in NDFs upon ALKBH3 knockdown. The data are presented as the mean± SD of triplicate experiments. Significance was determined by unpaired two-tailed Student's *t* test. ****p < 0.0001. (B) Western blot showing ALKBH3, COL1A1 and FN1 expression relative to GAPDH expression in NDFs upon ALKBH3 knockdown. (C) Densitometric analysis results showing the protein expression of ALKBH3, COL1A1 and FN1 relative to that of GAPDH in NDFs upon ALKBH3 knockdown. The data are presented as the mean ± SD of triplicate experiments. Significance was determined by unpaired two-tailed Student's t test. ****p < 0.0001. (D) A CCK-8 assay was used to evaluate the proliferation of NDFs upon ALKBH3 knockdown. The data are presented as the mean ± SD of triplicate experiments. Significance was determined by unpaired two-tailed Student's *t* test. (E) Apoptosis in NDFs with ALKBH3 knockdown was analysed by flow cytometry. All of the experiments were performed in triplicate. Significance was determined by unpaired two-tailed Student's *t* test. ***p < 0.001. (F) The cell cycle distribution of NDFs with ALKBH3 knockdown was analysed by flow cytometry. All of the experiments were performed in triplicate. (G) The migration of NDFs after ALKBH3 knockdown was analysed by a Transwell assay. Scale bar: 500 μm. All of the experiments were performed in triplicate, and five random fields were included in the analysis. Significance was determined by unpaired two-tailed Student's *t* test. HDF, normal human tissue-derived fibroblasts. (H) The long-term migratory ability of NDFs was evaluated with wound healing assays. Scale bar: 100 μm. All of the experiments were performed in triplicate, and 3 random fields at each time point were included in the analysis. Significance was determined by unpaired two-tailed Student's *t* test. ns, not significant.

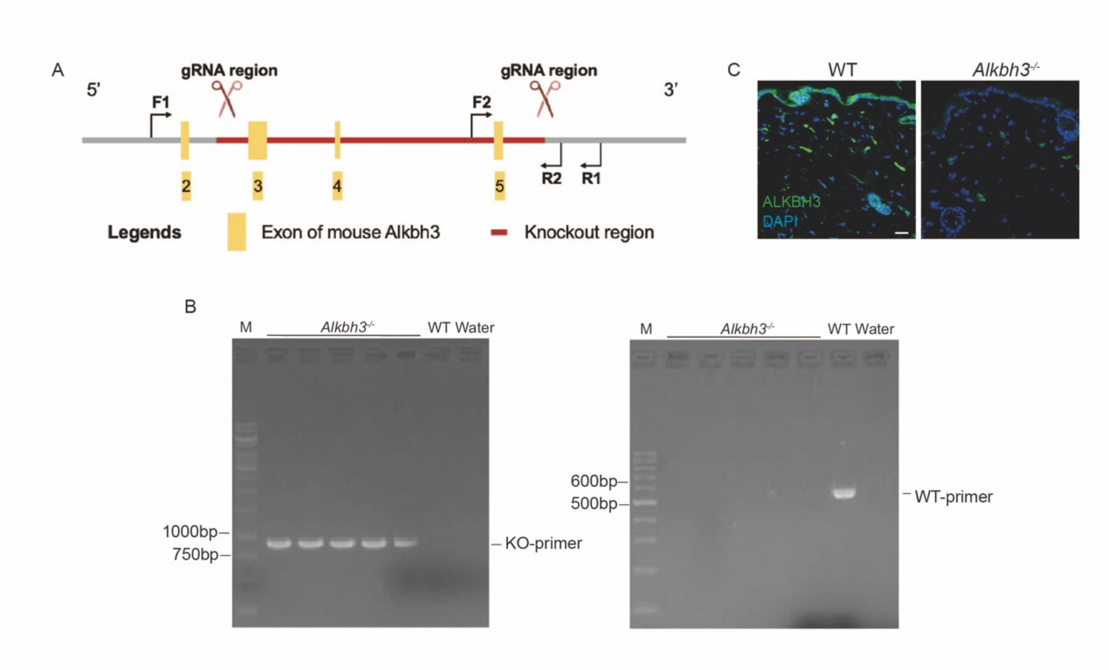

Figure S5. Validation of *Alkbh3⁻/⁻* mice

(A) Design of genotyping primers. F1R1=KO primer pair, F2R2=WT primer pair. (B) Genotyping of homozygous knockout (*Alkbh3*$^{-/-}$) and WT mice. (C) Immunofluorescence of ALKBH3 (green) and DAPI (blue) in the skin of WT and *Alkbh3*$^{-/-}$ mice. Scale bars: 20 μm.

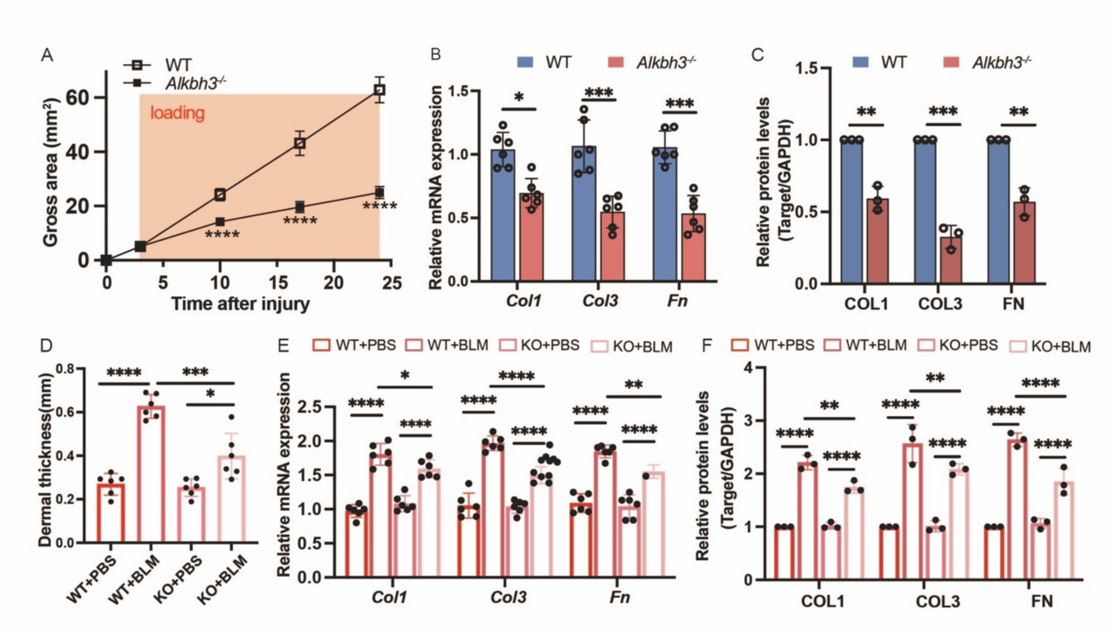

Figure S6. *Alkbh3$^{-/-}$* mice exhibited less severe skin fibrosis than did WT mice.

(A) Quantification of the scar area at all tested time points. two-tailed Student's *t* test, **** $p < 0.0001$. (B) qRT–PCR data showing *Col1a1* and *Fn1* expression in the mechanical stretch-induced HTS model. The data are presented as the mean± SD of triplicate experiments. two-tailed Student's *t* test, *$p < 0.05$, ***$p < 0.001$. (C) Densitometric analysis results showing the protein expression of COL1A1 and FN1 relative to that of GAPDH in the mechanical stretch-induced HTS model. The data are presented as the mean ± SD of triplicate experiments. two-tailed Student's t test, **$p < 0.01$. (D) Quantitative analysis of dermal thickness in the bleomycin-induced skin fibrosis model. two-tailed Student's *t* test, *$p < 0.05$, ***$p < 0.001$, ****$p < 0.0001$. (E) qRT–PCR data showing *Col1a1* and *Fn1* expression in the bleomycin-induced skin fibrosis model. The data are presented as the mean± SD of triplicate experiments. two-tailed Student's *t* test, *$p < 0.05$, ** $p < 0.01$, ****$p < 0.0001$. (F) Densitometric analysis results showing the protein expression of COL1A1 and FN1 relative to that of GAPDH in the bleomycin-induced skin fibrosis model. The data are presented as the mean ± SD of triplicate experiments. two-tailed Student's t test, ** $p < 0.01$. *** $p < 0.001$.

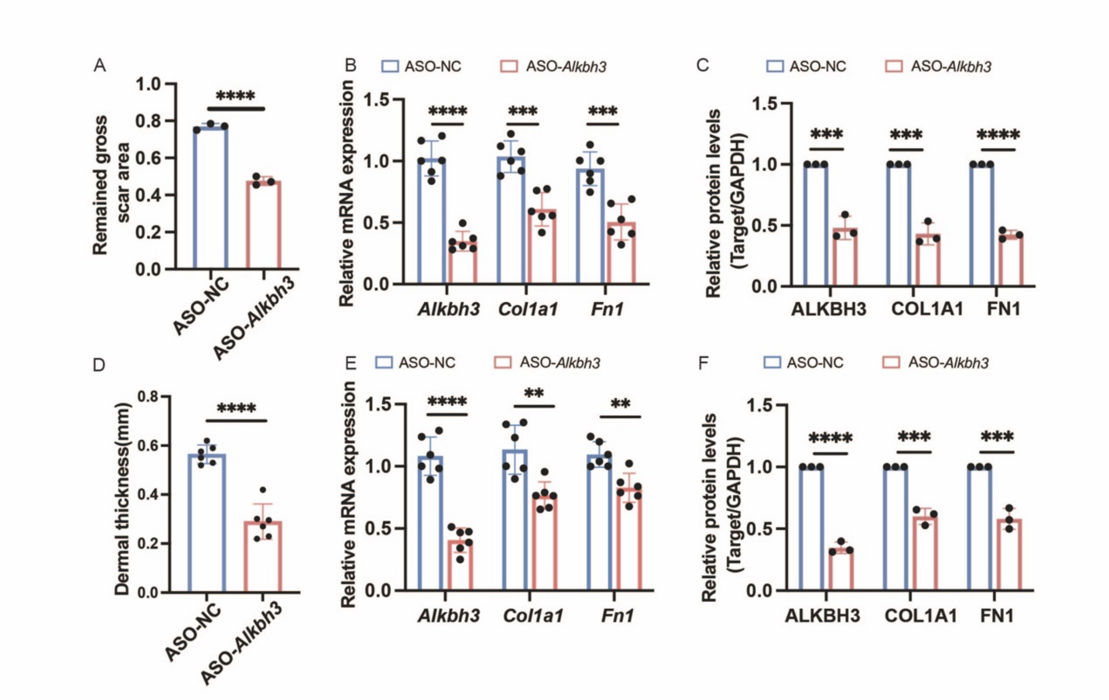

Figure S7. ASO-*Alkbh3* treatment promotes the progression of skin fibrosis *in vivo*

(A) Changes in the scar area before and after treatment with ASO-*Alkbh3*. two-tailed Student's *t* test, **** $p < 0.0001$. (B) qRT–PCR data showing *Alkbh3*, *Col1*a1 and *Fn1* expression in scar tissue obtained from mice in the two groups of mechanical stretch-induced HTS model. The data are presented as the mean± SD of triplicate experiments. two-tailed Student's *t* test, *** $p < 0.001$, **** $p < 0.0001$. (C) Densitometric analysis results showing the protein expression of ALKBH3, COL1A1 and FN1 relative to that of GAPDH in the two groups of mechanical stretch-induced HTS model. The data are presented as the mean ± SD of triplicate experiments. two-tailed Student's t test, ** $p < 0.01$. *** $p < 0.001$, **** $p < 0.0001$. (D) Quantitative analysis of dermal thickness in the two groups of bleomycin-induced skin fibrosis model. two-tailed Student's *t* test, **** $p < 0.0001$. (E) qRT–PCR data showing *Alkbh3, Col1a1* and *Fn1* expression in the two groups of bleomycin-induced skin fibrosis model. The data are presented as the mean± SD of triplicate experiments. two-tailed Student's *t* test, ** $p < 0.01$, **** $p < 0.0001$. (F) Densitometric analysis results showing the protein expression of ALKBH3, COL1A1 and FN1 relative to that of GAPDH in the two groups of bleomycin-induced skin fibrosis model. The data are presented as the mean ± SD of triplicate experiments. two-tailed Student's t test, *** $p < 0.001$, **** $p < 0.0001$.

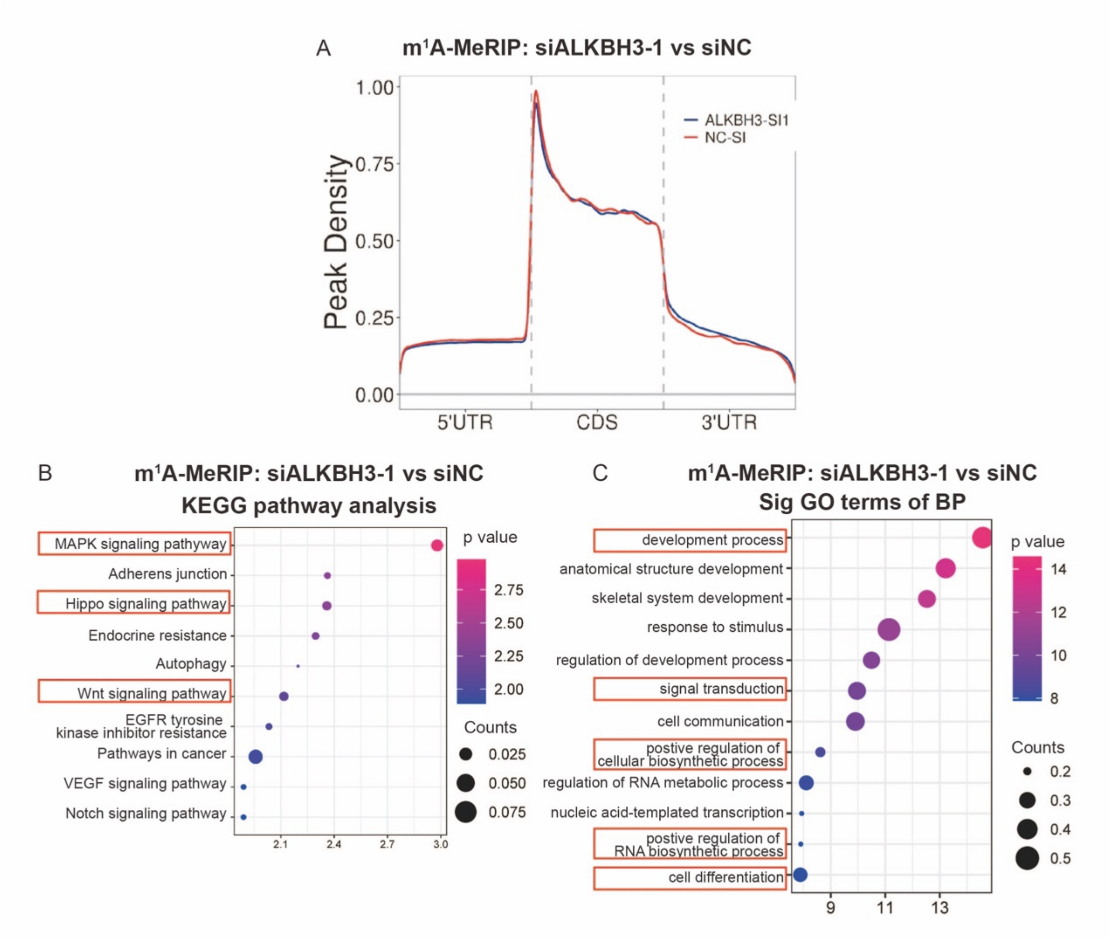

Figure S8. m^{1}A-modified genes are associated with fibrosis-related pathways.

(A) m^{1}A-meRIP-seq data showing the peak density of m^{1}A sites. Biological duplicates were analysed. (B) KEGG pathway analysis of m^{1}A-modified genes in wild-type and ALKBH3-deficient fibroblasts. (C) GO enrichment map of m^{1}A-regulated genes in wild-type and ALKBH3-deficient fibroblasts.

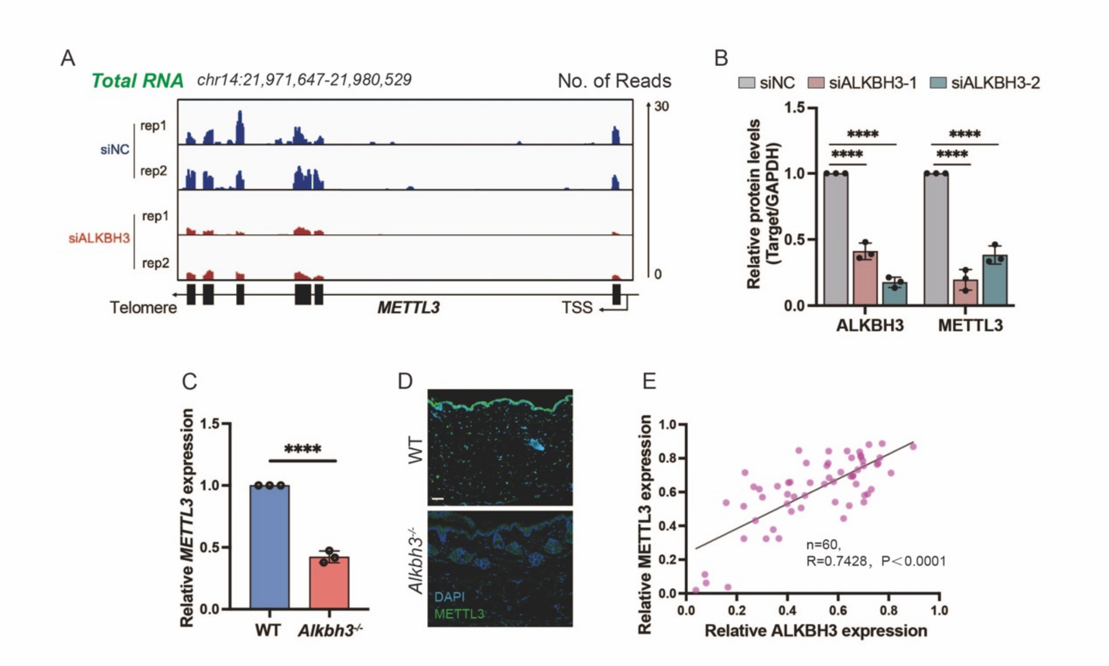

Figure S9. METTL3 expression is positively correlated with ALKBH3 expression. (A) IGV plot showing decreased *METTL3* expression in skin fibroblasts upon ALKBH3 knockdown. The experiments were performed in duplicate. (B) Densitometric analysis results showing the protein expression of ALKBH3 and METTL3 relative to that of GAPDH in HDFs upon ALKBH3 knockdown. The data are presented as the mean ± SD of triplicate experiments. two-tailed Student's t test, **** $p < 0.0001$. (C) qRT–PCR analysis of the mRNA expression of *Mettl3* in the skin of WT and *Alkbh3*$^{-/-}$ mice. The data are presented as the mean ± SD of triplicate experiments. Significance was determined by unpaired two-tailed Student's t test. ****$p < 0.0001$. (D) Immunofluorescence of METTL3 (green) and DAPI (blue) in WT and *Alkbh3*$^{-/-}$ mice. Scale bars: 50 μm. (E) Densitometric analysis results showing the correlation between ALKBH3 and METTL3 in HTS tissues (n=60, R=0.7428, p<0.0001).

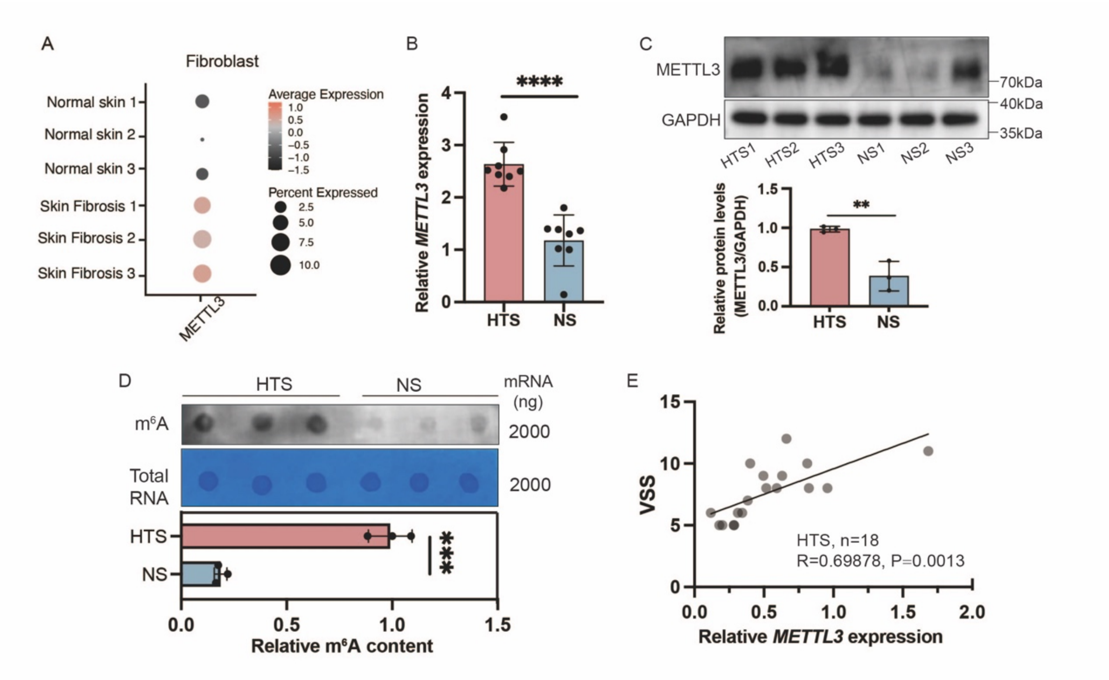

Figure S10. METTL3 expression is elevated in hypertrophic scars

(A) According to the single-cell analysis, METTL3 is highly expressed in fibroblasts from skin fibrosis compared to those from normal skin. (B) qRT–PCR data showing *METTL3* expression in HTS and NS samples. The data are presented as the mean ± SD of triplicate experiments. Significance was determined by unpaired two-tailed Student's t test. ****p < 0.0001. (C) Western blot showing METTL3 expression relative to GAPDH expression in HTS and NS samples. The data are representative of triplicate experiments. (D) Dot blot showing the m^{6}A signal relative to the methylene blue signal in HTS and NS samples. The data are presented as the mean ± SD of triplicate experiments. Significance was determined by unpaired two-tailed Student's t test. ***p < 0.001. (E) Correlation analysis between *METTL3* expression and the VSS score. *GAPDH* was used to normalize mRNA expression levels, and the $2^{-\Delta\Delta Ct}$ method was used to calculate the relative expression levels (n=18, R=0.69878, p =0.0013). The *ALKBH3* expression level in sample HTS1 was defined as 1, and the expression levels were normalized to the fold changes. HTS, hypertrophic scar; NS, normal skin; VSS, Vancouver Scar Scale

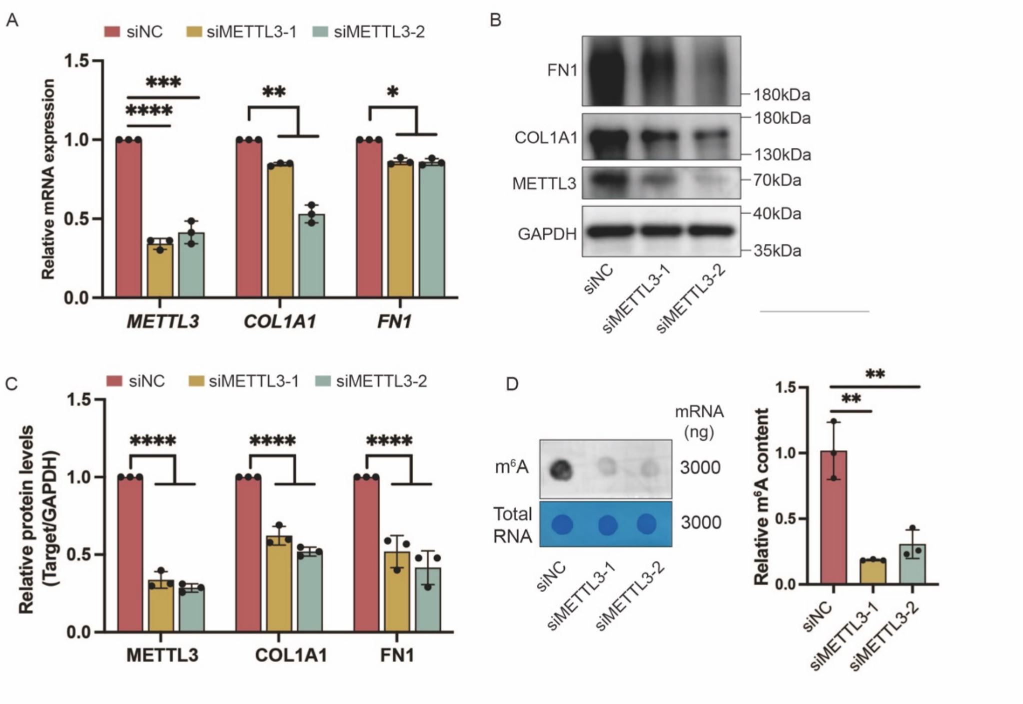

Figure S11. The m^{6}A methyltransferase METTL3 is critical for the collagen deposition function of fibroblasts

(A) qRT–PCR data showing *METTL3*, *COL1A1* and *FN1* expression in HDFs upon METTL3 knockdown. The data are presented as the mean± SD of triplicate experiments. Significance was determined by unpaired two-tailed Student's *t* test. * $p < 0.05$, ** $p < 0.01$, **** $p < 0.0001$. (B) Western blot showing METTL3, COL1A1 and FN1 expression relative to GAPDH expression in HDFs upon METTL3 knockdown. (C) Densitometric analysis results showing the protein expression of METTL3, COL1A1 and FN1 relative to that of GAPDH in HDFs upon METTL3 knockdown. The data are presented as the mean ± SD of triplicate experiments. Significance was determined by unpaired two-tailed Student's t test. **** $p < 0.0001$. (D) Dot blot showing the m^{6}A signal relative to the methylene blue signal in HDFs upon METTL3 knockdown. The data are presented as the mean ± SD of triplicate experiments. Significance was determined by unpaired two-tailed Student's t test. ** $p < 0.01$.

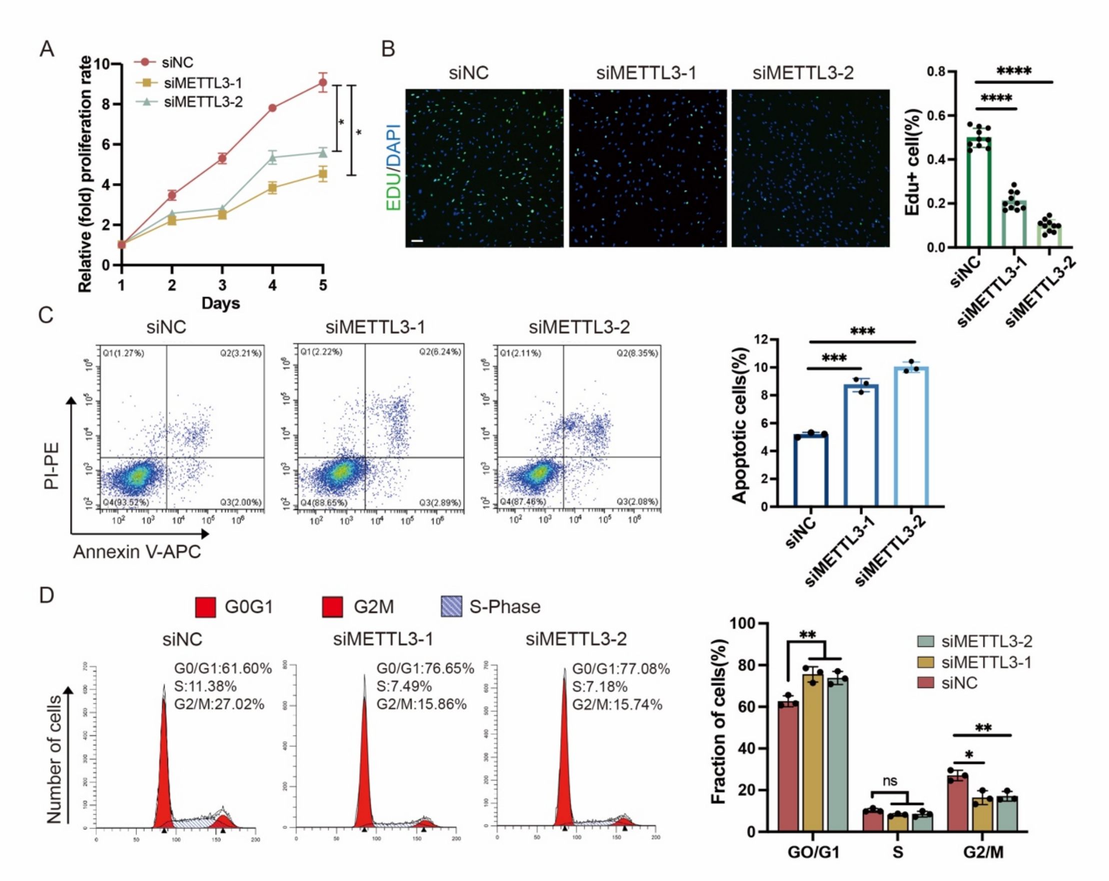

Figure S12. The m^{6}A methyltransferase METTL3 is critical for the growth capacity of fibroblasts

(**A**) A CCK-8 assay was used to evaluate the proliferation of HDFs upon METTL3 knockdown. The data are presented as the mean ± SD of triplicate experiments. Significance was determined by unpaired two-tailed Student's *t* test. * $p< 0.05$. (B) An EdU (green) incorporation assay was employed to evaluate the proliferation of HDFs upon METTL3 knockdown (scale bar=100 μm). The data are presented as the mean ± SD of triplicate experiments, and ten random fields were included in the analysis. Significance was determined by unpaired two-tailed Student's *t* test. ****$p < 0.0001$. (C) Apoptosis in METTL3-knockdown cells was analysed by flow cytometry. All of the experiments were performed in triplicate. Significance was determined by unpaired two-tailed Student's *t* test. *** $p < 0.001$. (D) Cell cycle distribution of HDFs following METTL3 knockdown. All of the experiments were performed in triplicate. Significance was determined by unpaired two-tailed Student's *t* test. * $p< 0.05$, ** $p< 0.01$.

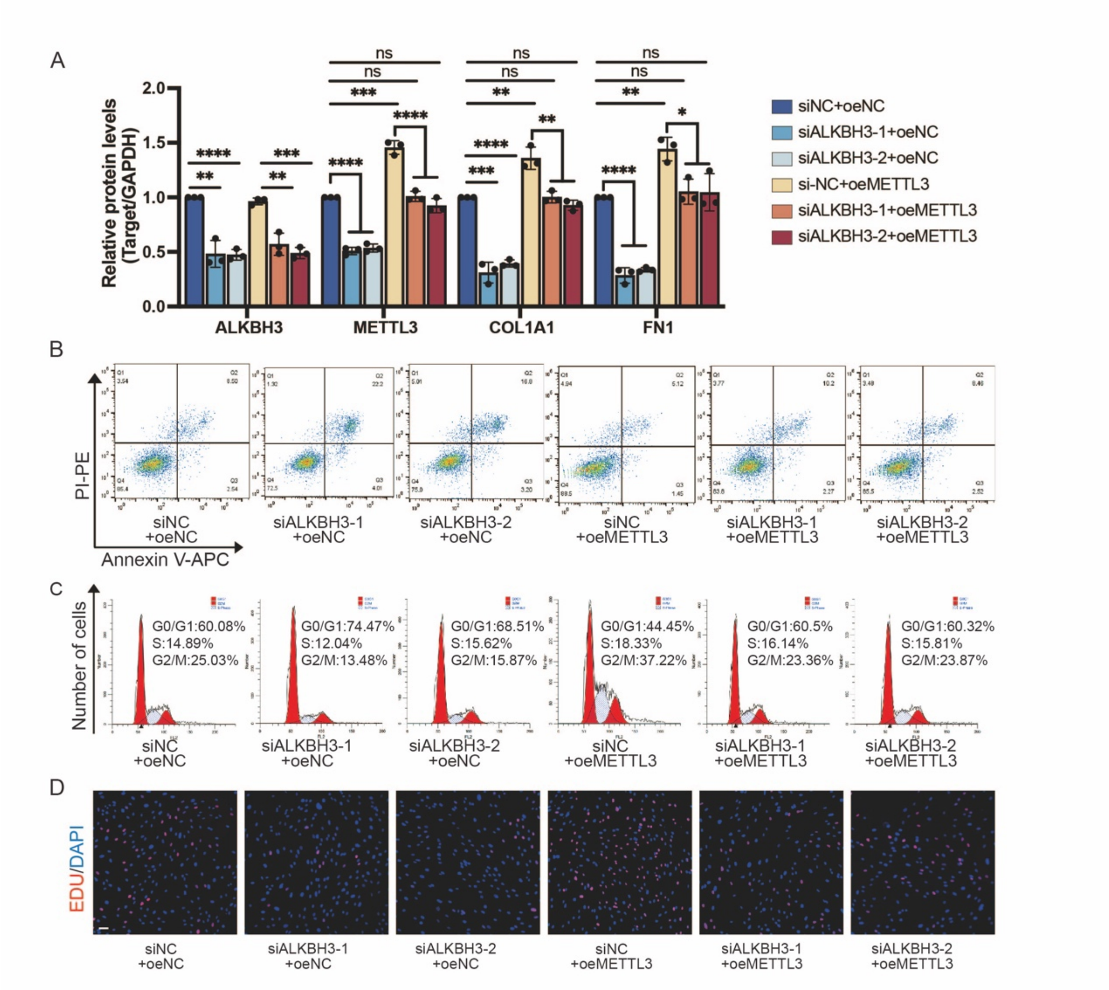

Figure S13. Exogenous overexpression of METTL3 counteracts the antifibrotic effects of ALKBH3 knockdown *in vitro*

(A). Densitometric analysis results showing the protein expression of METTL3, COL1A1 and FN1 relative to that of GAPDH in ALKBH3-deficient HDFs following METTL3 overexpression. The data are presented as the mean ± SD of triplicate experiments. Significance was determined by unpaired two-tailed Student's t test. * p<0.05, ** p<0.01, *** p<0.001, **** p<0.0001. (B) Apoptosis in ALKBH3-deficient HDFs following METTL3 overexpression was analysed by flow cytometry. All of the experiments were performed in triplicate. (C) The cell cycle distribution of ALKBH3-deficient HDFs following METTL3 overexpression was analysed by flow cytometry. All of the experiments were performed in triplicate. (D) The EdU (red) incorporation assay showed the effect of exogenous METTL3 overexpression on the proliferation of ALKBH3-deficient HDFs following METTL3 overexpression. Scale bar=50 μm. HDF, hypertrophic scar derived fibroblast.

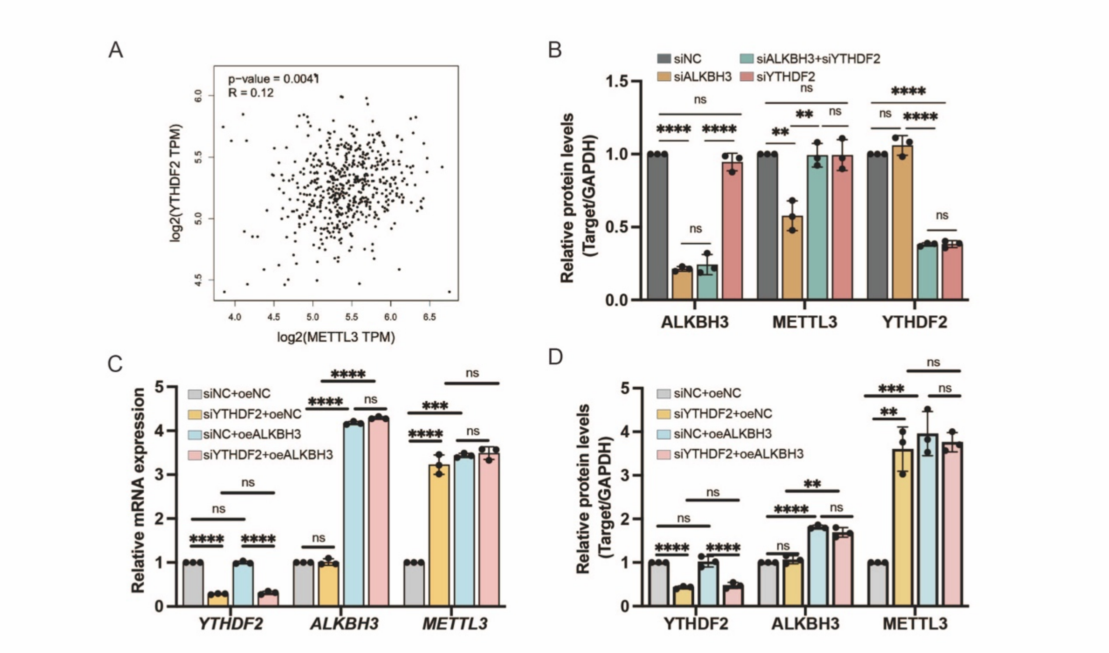

Figure S14. ***ALKBH3 regulates the expression of METTL3 in a YTHDF2-dependent manner***

(A) Correlation analysis of YTHDF2 expression and METTL3 expression in the GEPIA2 database. Significance was determined by *Pearson* correlation analysis (R = 0.12, $p = 0.0041$). (B) Densitometric analysis results showing the protein expression of ALKBH3, YTHDF2, and METTL3 relative to that of GAPDH in HDFs upon ALKBH3 and YTHDF2 knockdown. The data are presented as the mean ± SD of triplicate experiments. Significance was determined by unpaired two-tailed Student's t test. ** $p<0.01$, **** $p<0.0001$. (C) qRT–PCR data showing the mRNA expression of *YTHDF2*, *ALKBH3* and *METTL3* in ALKBH3-overexpressing HDFs with YTHDF2 knockdown. The data are presented as the mean± SD of triplicate experiments. Significance was determined by unpaired two-tailed Student's *t* test. ****$p < 0.0001$. (D) Densitometric analysis results showing the protein expression of YTHDF2, ALKBH3 and METTL3 relative to that of GAPDH in ALKBH3-overexpressing HDFs with YTHDF2 knockdown. The data are presented as the mean ± SD of triplicate experiments. Significance was determined by unpaired two-tailed Student's t test. **$p < 0.01$, ***$p < 0.001$, ****$p < 0.0001$.

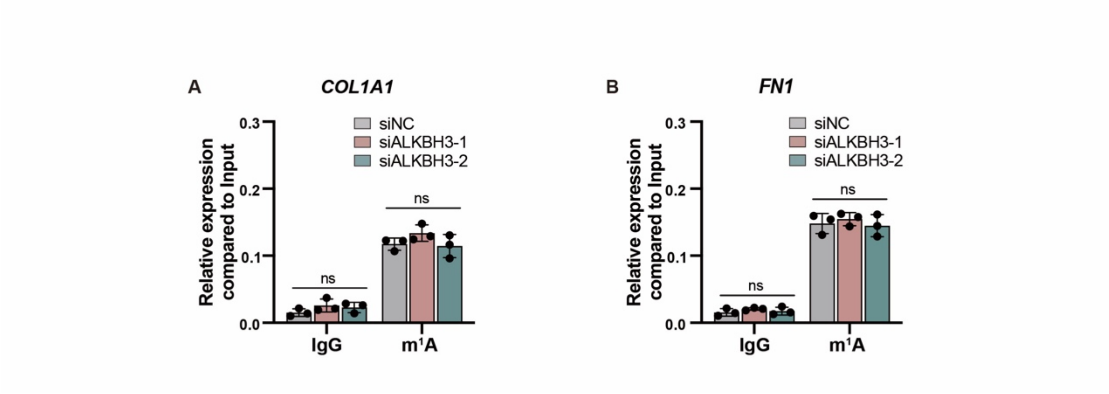

Figure S15. m^{1}A modification of *COL1A1* and *FN1* transcripts after ALKBH3 knockdown in HDFs.

(A) m^{1}A modification of *COL1A1* transcripts after ALKBH3 knockdown in HDFs was evaluated using m^{1}A-RIP-qPCR assays. The data are presented as the mean ± SD of triplicate experiments. Significance was determined by unpaired two-tailed Student's *t* test. (B) m^{1}A modification of *FN1* transcripts after ALKBH3 knockdown in HDFs was evaluated using m^{1}A-RIP-qPCR assays. The data are presented as the mean ± SD of triplicate experiments. Significance was determined by unpaired two-tailed Student's *t* test.

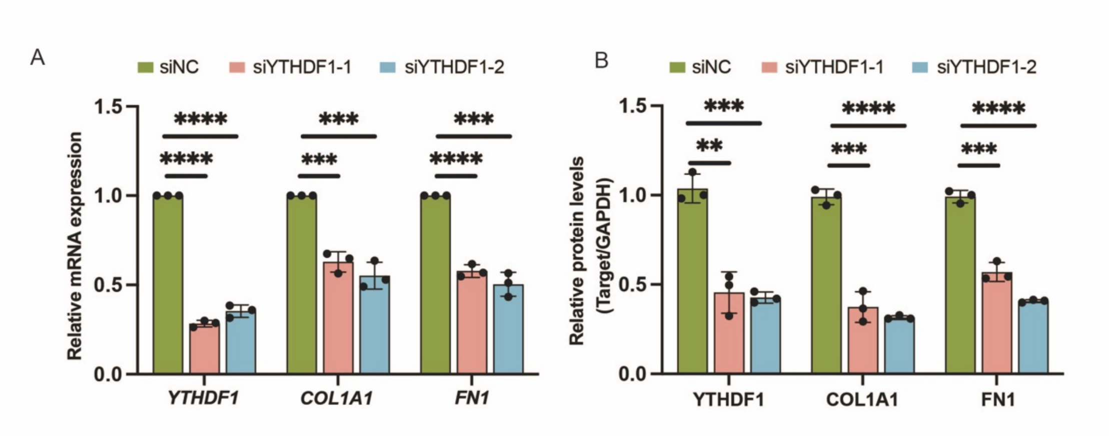

Figure S16. YTHDF1 reduces the expression of *COL1A1* and *FN1*.

(A) qRT–PCR data showing *YTHDF1*, *COL1A1* and *FN1* expression in HDFs upon YTHDF1 knockdown. The data are presented as the mean ± SD of triplicate experiments. Significance was determined by unpaired two-tailed Student's *t* test. *** $p < 0.001$, **** $p < 0.0001$. (B) Densitometric analysis results showing the protein expression of YTHDF1, COL1A1 and FN1 relative to that of GAPDH in HDFs upon YTHDF1 knockdown. The data are presented as the mean ± SD of triplicate

experiments. Significance was determined by unpaired two-tailed Student's t test. ** $p < 0.01$, *** $p < 0.001$, **** $p < 0.0001$.

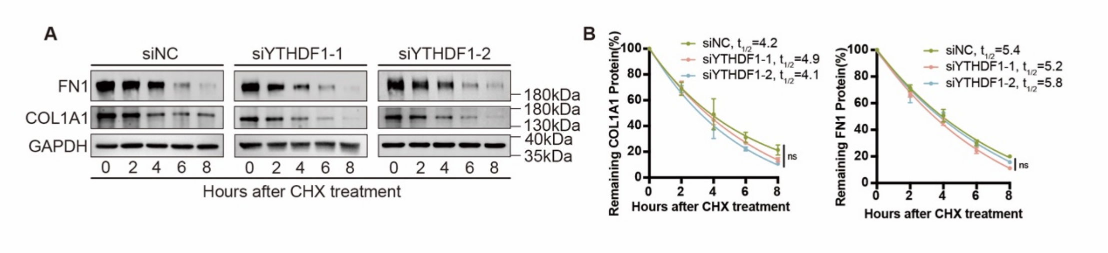

Figure S17. The protein half-life between YTHDF1 plasmid group and the control group.

(A) COL1A1 and FN1 protein expression following CHX (100 μg/mL) treatment in YTHDF1-silenced HDFs. (B) Densitometric analysis of the half-life of COL1A1 and FN1 in HDFs with YTHDF1 knockdown after CHX (100 μg/mL) treatment for 0, 2, 4, 6, or 8 h. Data are presented as the mean ± SD of triplicate experiments. Significance was determined by unpaired two-tailed Student's t test.

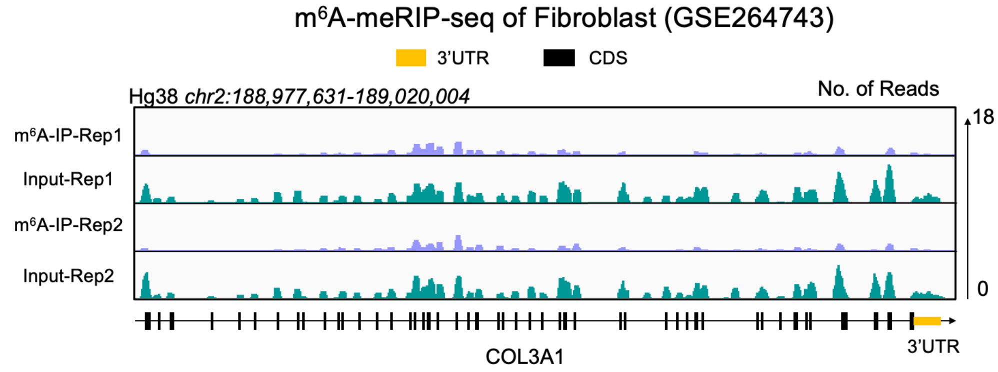

Figure S18. *COL3A1* is not affected by METTL3-mediated m^{6}A modification.

(A) IGV tracks from the m^{6}A-meRIP-seq analysis showing no enrichment of m^{6}A on *COL3A1* transcription.

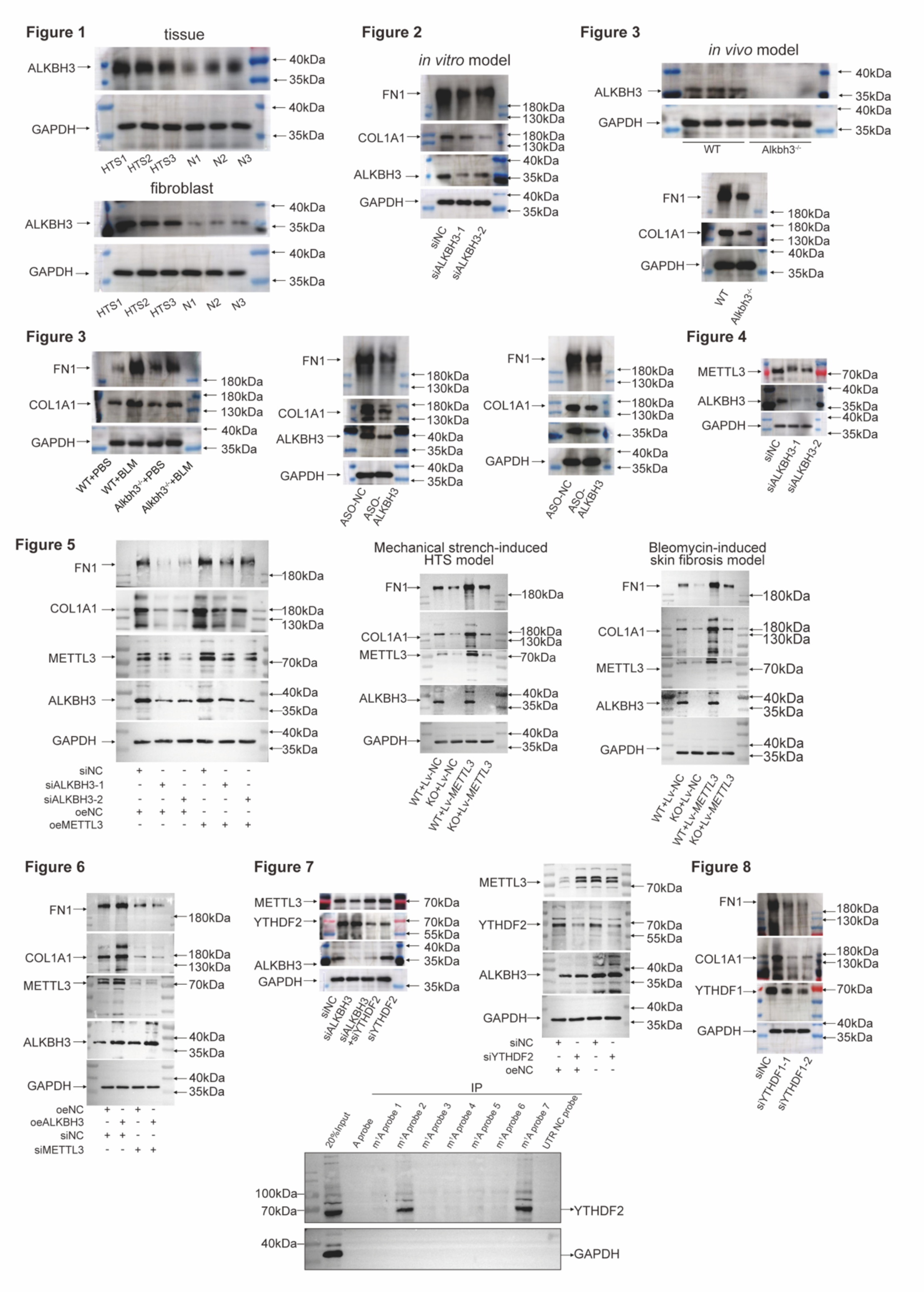

Figure S19. Uncropped original western blots.

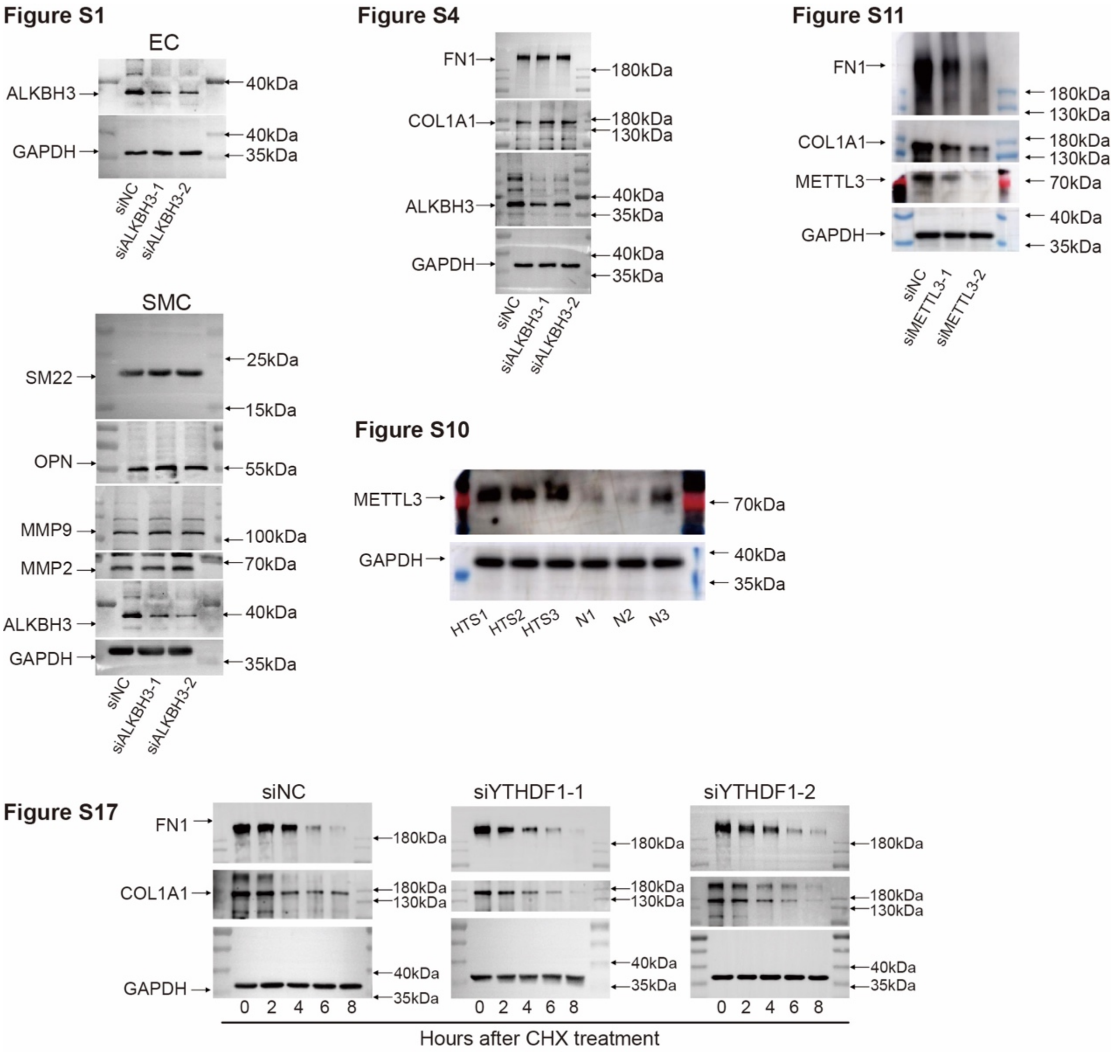

Figure S20. Uncropped original western blots.

Table S1. Vancouver Scar Scale

Items	Feature	Score
Vascularity	Normal	0
	Pink	1
	Red	2
	Purple	3
Pigmentation	Normal	0
	Hypopigmentation	1
	Mixed-pigmentation	2
	Hyperpigmentation	3
Pliability(elasticity)	Normal	0
	Supple(flexible with minimal resistance)	1
	Yielding(giving way to pressure	2
	Firm(inflexible, not easily moved, resistant to manual pressure)	3
	Banding(rope-like tissue that blanches with extension of the scar)	4
	Contracture(permanent shortening of scar, producing deformity or distortion	5
Height	Flat	0
	＜2mm	1
	2-5mm	2
	＞5mm	3
Pain	None	0
	Occasional	1
	Requires medication	2
Itchiness	None	0
	Occasional	1
	Requires medicaion	2

Table S2. Clinical and experimental information of patients whose samples were used in this study

Order	Sex	Age(y)	Body location	VSS	Experiments and analysis
HTS1 NS1	Female	24	Chest	12	qRT-PCR (Figure 1B, 1J, 4L, S10A, 10D), tissue microarray staining (Figure 4M), dotblot (1A, S10C)
HTS2 NS2	Male	22	Chest	10	qRT-PCR (Figure 1B, 1J, 4L, S10A, 10D); western blot (Figure 1G; S10B); tissue microarray staining (Figure 4M)
HTS3 NS3	Female	44	Abdomen	9	qRT-PCR (Figure 1B, 1J, 4L, S10A, 10D); tissue microarray staining (Figure 4M), dotblot (1A, S10C)
HTS4 NS4	Male	36	Face	9	qRT-PCR (Figure 1B, 1J, 4L, S10A, 10D); tissue microarray staining (Figure 4M), dotblot (1A, S10C)
HTS5 NS5	Female	29	Chest	11	qRT-PCR (Figure 1J, 4L, S10A, 10D); Immunofluorescence (Figure 1F); tissue microarray staining (Figure 4M)
HTS6 NS6	Female	34	Abdomen	8	qRT-PCR (Figure 1J, 4L, S10A, 10D); western blot (Figure 1G; S10B); tissue microarray staining (Figure 4M)
HTS7 NS7	Male	28	Chest	8	qRT-PCR (Figure 1J, 4L, S10A, 10D); western blot (Figure 1G; S10B); tissue microarray staining (Figure 4M)
HTS8	Female	52	Abdomen	8	qRT-PCR (Figure 1J, 4L, S10A, 10D); tissue microarray staining (Figure 4M)
HTS9	Female	27	Armpits	6	qRT-PCR (Figure 1J, 4L, S 10D); tissue microarray staining (Figure 4M)

HTS10	Male	31	Shoulder	5	qRT-PCR (Figure 1J, 4L, S 10D); tissue microarray staining (Figure 4M)
HTS11	Female	23	Perineum	7	qRT-PCR (Figure 1J, 4L, S 10D); tissue microarray staining (Figure 4M)
HTS12	Male	27	Neck	6	qRT-PCR (Figure 1J, 4L, S 10D); tissue microarray staining (Figure 4M)
HTS13	Female	23	Chest	8	qRT-PCR (Figure 1J, 4L, S 10D); tissue microarray staining (Figure 4M)
HTS14	Female	19	Finger	6	qRT-PCR (Figure 1J, 4L, S 10D); tissue microarray staining (Figure 54M)
HTS15	Male	40	Face	5	qRT-PCR (Figure 1J, 4L, S 10D); tissue microarray staining (Figure 4M)
HTS16	Male	51	Chest	5	qRT-PCR (Figure 1J, 4L, S 10D); tissue microarray staining (Figure 4M)
HTS17	Male	33	Chest	10	qRT-PCR (Figure 1J, 4L, S 10D); tissue microarray staining (Figure 4M)
HTS18	Female	28	Armpits	5	qRT-PCR (Figure 1J, 4L, S 10D); tissue microarray staining (Figure 4M)
HTS19	Male	34	Neck	11	tissue microarray staining (Figure 4M)
HTS20	Male	25	Face	9	tissue microarray staining (Figure 4M)
HTS21	Female	65	Chest	10	tissue microarray staining (Figure 4M)
HTS22	Female	32	Abdomen	8	tissue microarray staining (Figure 4M)
HTS23	Male	28	Neck	7	tissue microarray staining (Figure 4M)
HTS24	Male	39	Shoulder	8	tissue microarray staining (Figure 4M)
HTS25	Female	54	Chest	11	tissue microarray staining (Figure 4M)
HTS26	Male	62	Abdomen	5	tissue microarray staining (Figure 4M)

HTS27	Male	30	Chest	4	tissue microarray staining (Figure 4M)
HTS28	Male	69	Face	9	tissue microarray staining (Figure 4M)
HTS29	Female	23	Chest	10	tissue microarray staining (Figure 4M)
HTS30	Female	42	Face		tissue microarray staining (Figure 4M)
HTS31	Male	5	Abdomen		tissue microarray staining (Figure 4M)
HTS32	Female	63	Neck		tissue microarray staining (Figure 4M)
HTS33	Male	45	Shoulder		tissue microarray staining (Figure 4M)
HTS34	Male	23	Neck		tissue microarray staining (Figure 4M)
HTS35	Female	25	Chest		tissue microarray staining (Figure 4M)
HTS36	Male	26	Chest		tissue microarray staining (Figure 4M)
HTS37	Female	52	Face		tissue microarray staining (Figure 4M)
HTS38	Male	34	Abdomen		tissue microarray staining (Figure 4M)
HTS39	Female	31	Chest		tissue microarray staining (Figure 4M)
HTS40	Female	29	Chest		tissue microarray staining (Figure 4M)
HTS41	Male	39	Chest		tissue microarray staining (Figure 4M)
HTS42	Female	62	Abdomen		tissue microarray staining (Figure 4M)
HTS43	Female	34	Abdomen		tissue microarray staining (Figure 4M)
HTS44	Male	33	Back		tissue microarray staining (Figure 4M)
HTS45	Male	10	Neck		tissue microarray staining (Figure 4M)
HTS46	Female	12	Abdomen		tissue microarray staining (Figure 4M)
HTS47	Male	44	Neck		tissue microarray staining (Figure 4M)
HTS48	Male	64	Back		tissue microarray staining (Figure 4M)

HTS49	Male	22	Neck	tissue microarray staining (Figure 4M)
HTS50	Female	36	Chest	tissue microarray staining (Figure 4M)
HTS51	Female	9	Back	tissue microarray staining (Figure 4M)
HTS52	Female	45	Chest	tissue microarray staining (Figure 4M)
HTS53	Male	22	Abdomen	tissue microarray staining (Figure 4M)
HTS54	Female	49	Neck	tissue microarray staining (Figure 4M)
HTS55	Male	29	Chest	tissue microarray staining (Figure 4M)
HTS56	Female	61	Neck	tissue microarray staining (Figure 4M)
HTS57	Male	33	Chest	tissue microarray staining (Figure 4M)
HTS58	Male	56	Abdomen	tissue microarray staining (Figure 4M)
HTS59	Female	43	Abdomen	tissue microarray staining (Figure 4M)
HTS60	Female	21	Chest	tissue microarray staining (Figure 4M)
NS8	Male	56	Abdomen	qRT-PCR (Figure 5F)
NS9	Female	20	Neck	qRT-PCR (Figure 5F)
NS10	Female	32	Abdomen	qRT-PCR (Figure 5F)
NS11	Female	43	Chest	qRT-PCR (Figure 5F)
NS12	Male	47	Chest	qRT-PCR (Figure 5F)
NS13	Male	38	Face	qRT-PCR (Figure 5F)

Abbreviations: HTS, hypertrophic scar; NS, normal skin; y, year; VSS, Vancouver Scar Scale.

Table S3. Primers used in experiments

Primer sequences for qRT–PCR			
GAPDH	Human	Forward	5’– GGAGTCCACTGGCGTCTTCA–3’
		Reverse	5’– GTCATGAGTCCTTCCACGATACC–3’
ALKBH3	Human	Forward	5’– GAACCAAATCCTCACTGGCAC–3’
		Reverse	5’– GCGTGTGGCACCAAAACTTA–3’
FTO	Human	Forward	5’– TTGCATGGATGAGCCAGCTT –3’
		Reverse	5’– TCTCCAACCCTGTTGCACAT –3’
TRMT6	Human	Forward	5’– AGAGCCTACTGCAGGAAATAG –3’
		Reverse	5’– ATACGGGTGGATGGCTTCAC –3’
TRMT61a	Human	Forward	5’– TAATGCCAGGAGCTTTGGGG –3’
		Reverse	5’– ACCCATCAGACACCATGCAG –3’
COL1A1	Human	Forward	5’– GCTTGGTCCACTTGCTTGAA –3’
		Reverse	5’– TTTGGGAAGGAGTGGAGGG –3’
COL3A1	Human	Forward	5’– AGCCTGGTAAGAATGGTGCC –3’
		Reverse	5’– TCCTTGCCATCTTCGCCTTT –3’
FN1	Human	Forward	5’– CGGTGGCTGTCAGTCAAAG –3’
		Reverse	5’– AAACCTCGGCTTCCTCCATAA –3’
ACTA2	Human	Forward	5’– AAAAGACAGCTACGTGGGTGA –3’
		Reverse	5’– GCCATGTTCTATCGGGTACTTC –3’
METTL3	Human	Forward	5’– GTGATCGTAGCTGAGGTTCGT –3’
		Reverse	5’– GGGTTGCACATTGTGTGGTC –3’
MRPL34	Human	Forward	5’– TAGGGCCTGGAGATGGGAC –3’
		Reverse	5’– CTGGAGCCACCTGCCAC –3’
DTYMK	Human	Forward	5’– CCGGTGCCAAGGAGTTACAG –3’
		Reverse	5’– TGTCCTCATGGACAGCTTCG –3’
LY6K	Human	Forward	5’– CTGCGAGACAACGAGATCCA –3’
		Reverse	5’– GGGTCTAGGGGTTGTCACGG –3’
AURKAIP1	Human	Forward	5’– GTTCTGGGACCTTTCGCTCC –3’
		Reverse	5’– GGCGATGTGCTGTAAAGGGG –3’

RPL36A	Human	Forward	5’– GGAGATAAGAAGAGAAAGGGCCA –3’
		Reverse	5’– CCCCACAAGCGGAATTGTATTG –3’
MRPL54	Human	Forward	5’– AGTACCCTGAATGGCTGTTCG –3’
		Reverse	5’– GCCAGATGTTCTGTTTCCGC –3’
RPLP2	Human	Forward	5’– AAGATCTTGGACAGCGTGGG –3’
		Reverse	5’– CAAGCTTGCCAATACCCTGG –3’
CFL1	Human	Forward	5’– TCCGGAAACATGGCCTCC –3’
		Reverse	5’–CTTCTTCACCTCCTCTGGC–3’
DGCR6L	Human	Forward	5’– CACCAACCCACAGGAGCTG –3’
		Reverse	5’– CAGTCCCAGGGCTGCATTC –3’
RPS19BP1	Human	Forward	5’– GAGTACCGGAAGCGAGAGTG –3’
		Reverse	5’– TTCTGGCGCAAAATCTGCTG –3’
TCP1	Human	Forward	5’– CGCTCCCAAAACGGATGTAAC –3’
		Reverse	5’– TTGCAGATCAGCCAGCTCAC –3’
MRPL12	Human	Forward	5’– CAACCTCGTCCAGGCAAAGA –3’
		Reverse	5’– AACACAAGTCCTCCGAGCTG –3’
NIPSNAP1	Human	Forward	5’– CTCTGTGACGGCGCGTTTC –3’
		Reverse	5’– CTCCGTGAGGCTGTTGTAGG –3’
YTHDF1	Human	Forward	5’– CGACAACAAACCGGTCACAA –3’
		Reverse	5’– TTTCGACTCTGCCGTTCCTTG –3’
YTHDF2	Human	Forward	5’– GCCTCTTGGAGCAGTACAA –3’
		Reverse	5’– GCATTATTGGGCCTTGCCTG –3’
YTHDF3	Human	Forward	5’– CGAGGCCCTCATTTTGGGTT –3’
		Reverse	5’– CCTTGCCCTTTAGGTCTCTGA –3’
SM22	Human	Forward	5’–GTGGTGAAGTCATTTAAGACAAACG –3’
		Reverse	5’–GTTTGCCATGACTGTTCAGTGT –3’
OPN	Human	Forward	5’–AGCAGCTTTACAACAAATACCCAG –3’
		Reverse	5’–TTACTTGGAAGGGTCTGTGGG –3’
MMP2	Human	Forward	5’– CCCACTGCGGTTTTCTCGAAT–3’

		Reverse	5’– CAAAGGGGTATCCATCGCCAT–3’
MMP9	Human	Forward	5’– TGTACCGCTATGGTTACACTCG–3’
		Reverse	5’– GGCAGGGACAGTTGCTTCT–3’
Mettl3	Mouse	Forward	5’–CTTGCCATCTCTACGCCAGA–3’
		Reverse	5’–TCATGGCAGACAGCTTGGAG–3’
Alkbh3	Mouse	Forward	5’–GGACTCCTCGAACTGACAGC–3’
		Reverse	5’–AAGGGATGGCTACCTTGACG–3’
Gapdh	Mouse	Forward	5’–CAGTGGCAAAGTGGAGATTGTTG–3’
		Reverse	5’–TCGCTCCTGGAAGATGGTGAT–3’
Col1a1	Mouse	Forward	5’–CGATGGATTCCCGTTCGAGT–3’
		Reverse	5’–CGATCTCGTTGGATCCCTGG–3’
Fn1	Mouse	Forward	5’–GGCCACCATTACTGGTCTGG –3’
		Reverse	5’–GGAAGGGTAACCAGTTGGGG–3’
Primer sequences for RNA Binding Protein Immunoprecipitation (RIP)			
METTL3	Human	Forward	5’–GGTGTCCGCGTGAGAATTG–3’
		Reverse	5’–CACGTGTCCGACATCCTAGTC–3’
COL1A1	Human	Forward	5’–AGGTTGGGATGGAGGGAGTTTAC–3’
		Reverse	5’–GGACGTTGGTGCCCCAGAC–3’
FN1	Human	Forward	5’–GCGAAGGGAAGCAAACTTGG–3’
		Reverse	5’–ATTTGCTGAGCCTGCCTCTT–3’

Table S4. Antibodies used in experiments

Antibodies	Source	Identifier	Dilution
anti–ALKBH3	Proteintech	Cat No. 12292-1-AP	IF: 1:500; WB: 1:1000
anti-ALKBH3	Santa cruz	sc-376520	IF: 1:300; WB: 1:1000
anti–GAPDH	Proteintech	Cat No. 10494-1-AP	WB: 1:10000
anti–METTL3	Proteintech	Cat No. 15073–1–AP	IF: 1:500; WB: 1:1000
anti–COL1A1	Proteintech	Cat No. 14695-1-AP	WB: 1:2000
anti–COL3A1	Proteintech	Cat No. 22734-1-AP	WB: 1:1000
anti–FN1	Proteintech	Cat No. 15613-1-AP	WB: 1:1000
anti–ACTA2	Abcam	ab7817	IF: 1:300; WB: 1:2000
anti–m^1A	Abcam	ab208196	RIP: 5μg; DB: 1:1000
anti-m6A	abclonal	A19841	DB: 1:1000
anti–YTHDF1	Proteintech	Cat No. 17479–1–AP	RIP: 5μg; WB: 1:1000
anti–YTHDF2	Proteintech	Cat No. 24744–1–AP	RIP: 5μg; WB: 1:1000
anti–YTHDF3	Proteintech	Cat No. 25537–1–AP	RIP: 5μg; WB: 1:1000
anti-SM22	Proteintech	Cat No. 10493-1-AP	WB: 1:1000
anti-OPN	Proteintech	Cat No. 22952-1-AP	WB: 1:1000
anti-MMP2	Proteintech	Cat No. 10373-2-AP	WB: 1:1000
anti-MMP9	Proteintech	Cat No. 10375-2-AP	WB: 1:1000
Normal rabbit IgG antibody	Millipore	Cat#PP64	RIP: 5μg
Anti–rabbit IgG, HRP–linked Antibody	CST	#7074S	WB: 1:2500
Anti–mouse IgG (H+L), F(ab')2 Fragment (Alexa Fluor® 488 Conjugate)	CST	#4408S	IF: 1:400
Anti–rabbit IgG (H+L), F(ab')2 Fragment (Alexa Fluor® 594 Conjugate)	CST	#8889S	IF: 1:400
Anti–rabbit IgG (H+L), F(ab')2 Fragment (Alexa Fluor® 488 Conjugate)	CST	#4412	IF: 1:400
Anti–mouse IgG (H+L), F(ab')2	CST	#8890	IF: 1:400

Fragment (Alexa
Fluor® 594 Conjugate)

Table S5. Oligonucleotides used for siRNA expression vector

Oligonucleotides name	Sequence (5'–3')
ALKBH3–si1–sense	GGAUAUAACUUAUCAGCAATT
ALKBH3–si1–antisense	UUGCUGAUAAGUUAUAUCCTT
ALKBH3–si2–sense	CCUUACACUUAUUCAAGAATT
ALKBH3–si2–antisense	UUCUUGAAUAAGUGUAAGGTT
METTL3–si1–sense	GCAAGAAUUCUGUGACUAUTT
METTL3–si1–antisense	AUAGUCACAGAAUUCUUGCTT
METTL3–si2–sense	GCUGCACUUCAGACGAAUUTT
METTL3–si2–antisense	AAUUCGUCUGAAGUGCAGCTT
YTHDF2–si–sense	GGCUUUGGUUCAGAAUAUATT
YTHDF2–si–antisense	UAUAUUCUGAACCAAAGCCTT
YTHDF1–si–sense	GUUCGUUACAUCAGAAGGAUATT
YTHDF1–si–antisense	UAUCCUUCUGAUGUAACGAACTT

Table S6. Primers used for METTL3/ALKBH3 overexpression plasmid

Oligonucleotides name	Sequence (5'–3')
METTL3–forwad	gacaagcttgcggccgcgtcggacacgtggagctc
METTL3–reverse	CAGATCCTTCGCGGCCGCctataaattcttaggtttagagatgatacc
ALKBH3–forwad	agctagcgaattcggaGccaccATGgaggaaaaaagacggcgagc
ALKBH3–reverse	tgaacctccacctccctcgagccagggtgcccctcgag

Table S7. Oligonucleotides used for luciferase reporter gene.

Oligonucleotides name	Sequence (5'-3')
METTL3-5'UTR-WT	ATTTTCCGGT TAGCCTTCGG GGTGTCCGCG TGAGAATTGG CTATATCCTG GAGCGAGTGC TGGGAGGTGC TAGTCCGCCG CGCCTTATTC GAGAGGTGTC AGGGCTGGGA GACTAGGATG
METTL3-5'UTR-MUT1	ATTTTCCGGT TAGCCTTCGG GGTGTCCGCG TGAGTATTGG CTATATCCTG GAGCGAGTGC TGGGAGGTGC TAGTCCGCCG CGCCTTATTC GAGAGGTGTC AGGGCTGGGA GACTAGGATG

METTL3-5'UTR-MUT2	ATTTTCCGGT TAGCCTTCGG GGTGTCCGCG TGAGAATTGG CTATATCCTG GTGCGAGTGC TGGGAGGTGC TAGTCCGCCG CGCCTTATTC GAGAGGTGTC AGGGCTGGGA GACTAGGATG
METTL3-5'UTR-MUT3	ATTTTCCGGT TAGCCTTCGG GGTGTCCGCG TGAGAATTGG CTATATCCTG GAGCGAGTGC TGGGTGGTGC TAGTCCGCCG CGCCTTATTC GAGAGGTGTC AGGGCTGGGA GACTAGGATG
METTL3-5'UTR-MUT4	ATTTTCCGGT TAGCCTTCGG GGTGTCCGCG TGAGAATTGG CTATATCCTG GAGCGAGTGC TGGGAGGTGC TAGTCCGCCG CGCCTTATTC GAGTGGTGTC AGGGCTGGGA GACTAGGATG
METTL3-5'UTR-MUT5	ATTTTCCGGT TAGCCTTCGG GGTGTCCGCG TGAGAATTGG CTATATCCTG GAGCGAGTGC TGGGAGGTGC TAGTCCGCCG CGCCTTATTC GAGAGGTGTC AGGGCTGGGTGACTAGGATG
METTL3-5'UTR-MUT6	ATTTTCCGGT TAGCCTTCGG GGTGTCCGCG TGAGAATTGG CTATATCCTG GAGCGAGTGC TGGGAGGTGC TAGTCCGCCG CGCCTTATTC GAGAGGTGTC AGGGCTGGGAGACTAGGTTG
METTL3-5'UTR-MUT1-6	ATTTTCCGGT TAGCCTTCGG GGTGTCCGCG TGAGTATTGG CTATATCCTG GTGCGAGTGC TGGGTGGTGC TAGTCCGCCG CGCCTTATTC GAGTGGTGTC AGGGCTGGGTGACTAGGTTG

Table S8. ssRNA probes used for RNA pull–down assay

Oligonucleotides name	Sequence (5'–3')
A probe	Biotin-ATTTTCCGGT TAGCCTTCGG GGTGTCCGCG TGAGAATTGG CTATATCCTG GAGCGAGTGC TGGGAGGTGC TAGTCCGCCG CGCCTTATTC GAGAGGTGTC AGGGCTGGGA GACTAGGATG
m^1A probe 1	Biotin-ATTTTCCGGT TAGCCTTCGG GGTGTCCGCG TGAGm^1AATTGG CTATATCCTG GAGCGAGTGC TGGGAGGTGC TAGTCCGCCG CGCCTTATTC GAGAGGTGTC AGGGCTGGGA GACTAGGATG
m^1A probe 2	Biotin-ATTTTCCGGT TAGCCTTCGG GGTGTCCGCG TGAGAATTGG CTATATCCTG Gm^1AGCGAGTGC

	TGGGAGGTGC TAGTCCGCCG CGCCTTATTC GAGAGGTGTC AGGGCTGGGA GACTAGGATG
m^{1}A probe 3	Biotin-ATTTTCCGGT TAGCCTTCGG GGTGTCCGCG TGAGAATTGG CTATATCCTG GAGCGAGTGC TGGGm^{1}AGGTGC TAGTCCGCCG CGCCTTATTC GAGAGGTGTC AGGGCTGGGA GACTAGGATG
m^{1}A probe 4	Biotin-ATTTTCCGGT TAGCCTTCGG GGTGTCCGCG TGAGAATTGG CTATATCCTG GAGCGAGTGC TGGGAGGTGC TAGTCCGCCG CGCCTTATTC GAG m^{1}AGGTGTC AGGGCTGGGA GACTAGGATG
m^{1}A probe 5	Biotin-ATTTTCCGGT TAGCCTTCGG GGTGTCCGCG TGAGAATTGG CTATATCCTG GAGCGAGTGC TGGGAGGTGC TAGTCCGCCG CGCCTTATTC GAGAGGTGTC AGGGCTGGGm^{1}AGACTAGGATG
m^{1}A probe 6	Biotin-ATTTTCCGGT TAGCCTTCGG GGTGTCCGCG TGAGAATTGG CTATATCCTG GAGCGAGTGC TGGGAGGTGC TAGTCCGCCG CGCCTTATTC GAGAGGTGTC AGGGCTGGGAGACTAGGm^{1}ATG
m^{1}A probe 7	Biotin-ATTTTCCGGT TAGCCTTCGG GGTGTCCGCG TGAGm^{1}AATTGG CTATATCCTG Gm^{1}AGCGAGTGC TGGGm^{1}AGGTGC TAGTCCGCCG CGCCTTATTC GAGm^{1}AGGTGTC AGGGCTGGGm^{1}AGACTAGGm^{1}ATG
